# Systematic Analysis of the Genetic Variability That Impacts SUMO Conjugation and Their Involvement in Human Diseases

**DOI:** 10.1038/srep10900

**Published:** 2015-07-08

**Authors:** Hao-Dong Xu, Shao-Ping Shi, Xiang Chen, Jian-Ding Qiu

**Affiliations:** 1 Department of Chemistry, Nanchang University, Nanchang 330031, P.R.China; 2 Department of Mathematics, Nanchang University, Nanchang 330031, P.R.China; 3 Department of Materials and Chemical Engineering, Pingxiang College, Pingxiang 337055, P.R.China

## Abstract

Protein function has been observed to rely on select essential sites instead of requiring all sites to be indispensable. Small ubiquitin-related modifier (SUMO) conjugation or sumoylation, which is a highly dynamic reversible process and its outcomes are extremely diverse, ranging from changes in localization to altered activity and, in some cases, stability of the modified, has shown to be especially valuable in cellular biology. Motivated by the significance of SUMO conjugation in biological processes, we report here on the first exploratory assessment whether sumoylation related genetic variability impacts protein functions as well as the occurrence of diseases related to SUMO. Here, we defined the SUMOAMVR as sumoylation related amino acid variations that affect sumoylation sites or enzymes involved in the process of connectivity, and categorized four types of potential SUMOAMVRs. We detected that 17.13% of amino acid variations are potential SUMOAMVRs and 4.83% of disease mutations could lead to SUMOAMVR with our system. More interestingly, the statistical analysis demonstrates that the amino acid variations that directly create new potential lysine sumoylation sites are more likely to cause diseases. It can be anticipated that our method can provide more instructive guidance to identify the mechanisms of genetic diseases.

Cellular pathways involved in determining the fate of essential proteins through various post-translational modification (PTMs) events have become an increasingly important area of research^1^,^2^,^3^ and among these PTMs; a recently identified type of modification is small ubiquitin-related modifier (SUMO) conjugation or sumoylation^4^. As one of the most significant reversible PTMs of proteins, it has been studied to be involved in the various vital biologic processes, such as transcriptional regulation and signaling transductions^5^,^6^,^7^. Although sumoylation appears to involve only a small proportion of a target protein, the effects can be dramatic. Increasing evidences have been indicated that subtle changes of the state of the sumoylation could also affect sub nuclear targeting^8^, chromosome segregation^9^, the structural maintenance of various proteins, formation of the stable chromatin structure and many indispensable roles in mitosis^10^. Additionally a growing number of diseases are more likely to have closely link with the sumoylation process, including Alzheimer’s, Parkinson’s, familial amyotrophic sclerosis (FALS), cancer^8^ and diabetes^11^. Nonetheless, considering the young age of the field, there are large body of published works until now focusing more on the roles of SUMO in transcription, DNA repair, nuclear bodies and nucleocytoplasmic transport and no more to explore the complex relationship with those diseases in a deeper level^4^, which could help us gain profound understanding about the underlying connection between sumoylation and the mechanism of many disease.

Genetic variability, as the most common type of event in the human body, has been reported to be involved in a considerable number of human diseases^12^,^13^,^14^,^15^,^16^. Therefore, we here on the first exploratory assess whether sumoylation related genetic variability impacts protein functions as well as the occurrence of diseases related to SUMO. At present, rapid advances in genomic sequencing and the applications of innovative biotechnology have made it available to investigate complex phenomena in biological system, which impart a new impulse to the study of the role of genetic variation in susceptibility to disease. The most common type of genetic variation between individuals is single nucleotide polymorphisms (SNP)^17^. Although most of the genetic variations are considered to be neutral and harmless^18^, no synonymous single nucleotide polymorphism (nsSNP), accounting for nearly half of the known genetic variations linked to human inherited diseases, which occur in a region of coding gene may lead to the replacements of amino acids in corresponding protein product^19^,^20^. Since this type of variation can have a profound effect on human functions related to various disease. Understanding genetic variation in the context of human diseases thus holds the promise for “personalized medicine.” Therefore, proteome-wide analysis of nsSNP that affects sumoylation sites is an effective way of estimating how variation can affect function at a system level. The finding and functional characterization of biologically significant nsSNP is advancing our knowledge of genetic determinants for multifactorial disease^21^,^22^.

The amino acid substitution might alter the physicochemical property of a wild-type amino acid which would have impacts on the stability and dynamics of protein, disrupt the interacting interface and prohibit the protein to interact with other proteins^23^,^24^. Alternatively, by way of altering the types of residues of the target sites or key flanking residues, amino acid mutations could also influence PTMs of proteins^25^. For example, Ryu *et al.* and Ren *et al.* systematically investigated amino acid mutations from the perspective of phosphorylation, both of them proposed that a considerable amount of the amino acid mutations might affect the status of phosphorylation of protein as well as participate in rewiring biological pathways which associated with potential causation of various diseases^26^,^27^. We further performed a proteome-wide analysis of amino acid variations about the potential impact on protein lysine acetylation characteristics in human variant proteins and made a tentative exploration about what functional implication of genetic variations is in regard to cellular pathways of lysine acetylation^28^. Therefore, the addition or removal of sumoylation sites or the mutations occurred on the evolutionarily conserved sites adjacent to sumoylation sites through sumoylation related amino acid variations (SUMOAMVR) may also result in functional alterations in proteins that can lead to various diseases. For example, Justo *et al.* have investigated the relationship between genetic variability in USPL1 (ubiquitin-specific peptidase-like 1) that was identified as a SUMO isopeptidase and involved in the regulation of sumoylation and breast cancer and further drew a conclusion that functional nsSNPs in USPL1 called “rs7984952” which are implicated in amino acid exchanges from leucine to serine at position 531 was associated with risk for grade-3 breast tumors^29^. Besides, many researches have been reported that genetic variability may affect expression and activity of UBC9 which encodes a protein that conjugates SUMO to target proteins may have an impact on breast tumor progression^30^. In this regard, through comprehensive analysis of effect of the genetic mutations on the process of sumoylation in protein could enable us profoundly understand how genetic variability is involved in regulating biological processes and how they affect susceptibility to diseases. To efficiently accelerate the development of the underlying functional influence that genetic variability that impacts SUMO conjugation and their involvement in human diseases, an integrated platform combining experimentally data querying and unknown data annotation is highly demanded. Here, we developed a platform which provides a computational tool to efficiently and reliably identify the potential SUMOAMVRs for further experimental investigation. In this work, we report on the first exploratory assessment whether sumoylation related genetic variability impacts protein functions and the occurrence of diseases related to SUMO.

## Results

### Development of SumoPred for the Prediction of Lysine Sumoylation Sites

For systematically analyzing the lysine sumoylation and their involvement in related diseases, a prerequisite is to establish a comprehensive and reliable dataset. However, the experimental identification of sumoylated substrates is still labor-intensive and time-consuming, while only a small number of known sumoylation sites were collected. Thus, computational prediction of sumoylation sites from protein primary sequences, structural and evolutionary information can serve as an alternative solution. Therefore, we developed a prediction tool named SumoPred to determine whether a sequence which contains lysine residues can be sumoylated or not. The data sets of sumoylation sites were collected from several public databases, including UniProtKB/Swiss-Prot, PhosphoSite-Plus^31^, and HPRD databases^32^, etc. In total, these data sets contained 752 unique sumoylation sites and 19202 non-sumoylation sites by wiping off homologous fragments with a sensitive cutoff of 0.3. In the SumoPred, a local sliding window size of a maximum number of 10 residues flanking each lysine was chosen because the fragments of this size could cover the 10-amino acid NDSM region located downstream from the consensus motif-ΨKxE (where Ψ is a large aliphatic branched hydrophobic amino acid and x is any amino acid)^33^. In order to explore a comprehensive sumoylation site prediction tool, not only the biological characteristics of sequence-derived, including physicochemical properties of amino acids, the composition of K-spaced Amino Acid Pairs (CKSAAP) (i.e., pairs that are separated by k other amino acids), but also the structural characters of the fragments, which contain Average Accessible Surface Area (AASA) and the secondary structure of proteins, and also evolutionary information of amino acids termed Position Specific Scoring Matrix Profiles (PSSM) were investigated. Since these encoding contain a large amount of features vector, to avoid the potential over-fitting problem, it is worth picking up most important features by using a feature selection method known as F-select^34^. Finally, those above five encodings schemes further optimized have been utilized as the input vector to represent features from sumoylated and non- sumoylated fragments. We repeated the training procedures 10 times by randomly selecting the balanced negative samples from all of non-sumoylated fragments because of the significant imbalanced ratio of sumoylation sites to non-sumoylation sites. More details about the methods (such as analysis and selection of the feature, SVM training parameters, evaluation of the model, etc.) were described in the methodology and the Supporting Information.

### Evaluation and Comparison of the Lysine Sumoylation Prediction Model

**Figure 1:**
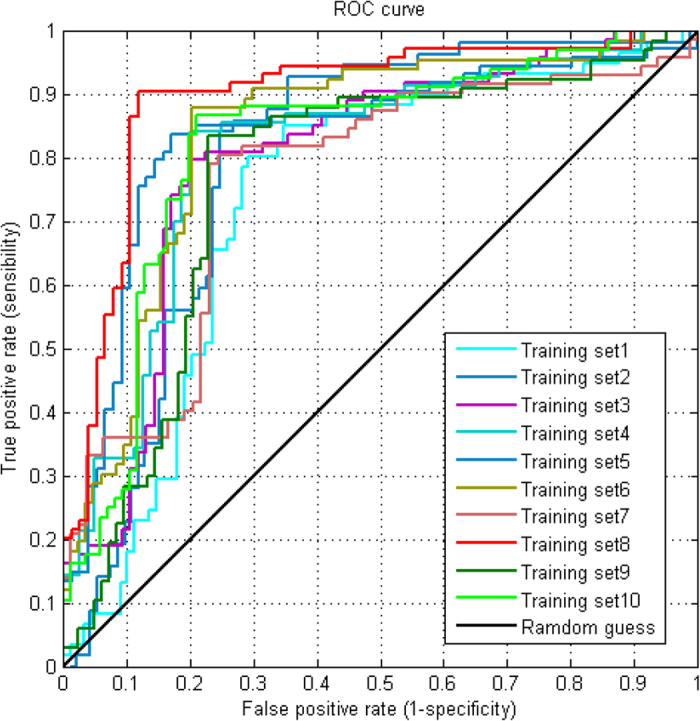
The receiver operating characteristic (ROC) curves of SumoPred prediction on 10 training sets.

In order to demonstrate the superiority of SumoPred, an independent test was conducted to compare the performance of our method with other previously published methods. We manually collected instances of experimentally identified sumoylation sites from the latest publications and omitted those sites that are listed in training sets to avoid overestimating the performance. Finally, the independent dataset is consisted of an additional 33 sumoylation sites and 721 non-sumoylation sites in 22 proteins. A summary of all the tools for sumoylation-site prediction was listed in Table S1. Among them, SUMOplot, 2006, was the first step in development of computational server for the prediction of sumoylation sites but had a bias of ΨKxE sites in the data. SUMOpre, 2008^35^, was developed on the basis of sequence data only with the probabilistic model of prediction. SSPFS, 2009^36^, employed mRMR and nearest neighbor algorithm trained on seven optimal amino acid properties selected from hundreds of amino acid properties. Moreover, seeSUMO, 2010^37^, was introduced as a web server using random forest-based algorithm for training, while SUMOhydro, 2012^38^, combined amino acid hydrophobicity and binary encoding scheme to predict sumoylation sites. GPS-SUMO, 2014^39^, is an updated version of SUMOsp^40^ and SUMOsp 2.0^41^ by improving the prediction algorithm, which applies GPS and MotifX on sumoylated site prediction. Six of above tools provide online prediction. Because SUMOsp 2.0 was demonstrated to be better than SUMOplot and SUMOpre^41^, and SUMOhydro did not support the batch prediction for multiple sequences, here we only compared SumoPred with GPS-SUMO and SeeSUMO. As shown in Table 1, after going through test, we acquired almost balanced sensitivity and specificity of 87.88% and 81.82% with our prediction system, respectively. When testing the independent set with the SeeSUMO of high stringency value, a high specificity (93.94%) was obtained, but a low sensitivity (9.09%). When with the low stringency value, the sensitivity of SeeSUMO was 90.91%, but the specificity was only 48.48%. Compared to GPS-SUMO, our method could offer good specificity as well as high sensitivity with all different stringency values. The MCC (the measure of the overall performance of biased datasets) value of SumoPred could attain to 0.6985 that is higher than the other two methods, which also demonstrate that our approach outperformed the GPS-SUMO and SeeSUMO. Besides, many existing methods are only considered one feature to build prediction models, so some other potential information will inevitably lose. Here, we have considered different aspects of the features (sequence-derived, structural characters, evolutionary information) when we build the model, so that our system can provide a more comprehensive prediction performance comparing with existing methods. Furthermore, the receiver operating characteristic (ROC) curves are drawn in Fig. 1 and the corresponding value of average area under the curve (AUC) was 84.67%. Since the prediction performance of different training sets is stable for the prediction of sumoylation sites, it is evident that the method is a robust predictor. In short, the performance of our model is reasonably good.

### Prediction of SUMOAMVR

**Figure 2:**
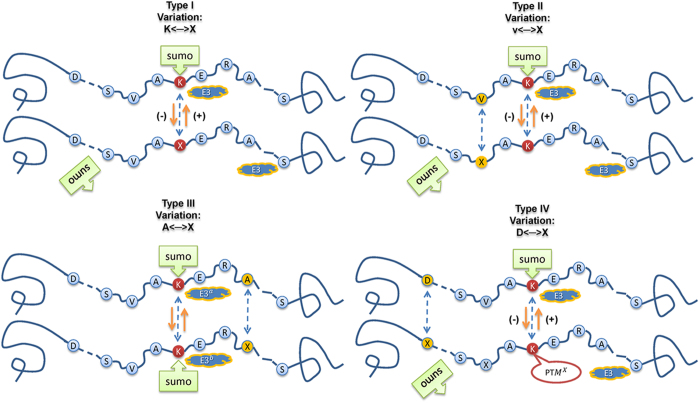
Schematic illustration of four of SUMOAMVRs, which include the change of an amino acid by lysine residue to create a potential new (Type I (+)) or remove an original lysine sumoylation site (Type I (−)); variations adjacent to sumoylation sites to create (Type II (+)) or remove (Type II (−)) sumoylation sites; and variations which may change the types of E3 ligase that recognize sumoylation sites, without changing the sumoylation site itself (Type III); variations adjacent to sumoylation sites and further transform the type of PTMs that it should have happened (Type IV). Yellow amino acid residues are mutation residues, sumo represents the small ubiquitin-related modifier, and a lysine (K) linked with a sumo represents that this K can be sumoylated by E3 ligase. E3^a^ represents one type of lysine E3 ligase and E3^b^ is another type of lysine E3 ligase.

The information of human genetic variations was extracted from the SwissVariant database (2014_02). We collected total 12512 human variant proteins containing 68779 amino acid variations, of which including 24399 diseases’ variants, 37878 polymorphic and 6502 unclassified variants. These mutations are considered more likely to be involved in functional and causal effect in diseases, although biochemical confirmations of such causal effects were only available for some of the variants. On the basis of these data, we further exploited the SwissVariant, UniProtKB/Swiss-Prot, PhosphoSite-Plus, HPRD and a number of other databases about their underlying effects as well as the related references about these variations and the corresponding sumoylation information. With the SumoPred, the SUMOAMVR could be identified when the predicted results of sumoylation sites were altered between the original and variant sequences. In other words, if the status of sumoylation site of specific protein substrate is altered due to change of the residue types of the target sites or one or some key flanking amino acids in this fragment in the before and after mutagenesis, then we define these mutations are SUMOAMVR related. All SUMOAMVRs were classified into four types according to the consequences of their influence on the sumoylation sites which were defined as follows (Fig. 2):(i)Type I, an amino acid variation occurs at a sumoylation position that directly adds (Type I (+)) or removes (Type I (−)) the sumoylation site;(ii)Type II, the amino acid variations not occur at sumoylation positions but on the adjacent positions of sumoyaltion sites that add (Type II (+)) or remove (Type II(−)) the sumoylation sites; (iii) Type III SUMOAMVR are caused by changes in the types of E3 ligase involved, rather than in the sumoylation sites itself, regardless of the positions of the variations, that is to say, before the amino acid variation adjacent to sumoylation site occurs, the first kind of E3 ligase(expressed as E3^a^ in Fig. 2) catalyzes lysine (K) and sumoylaiton happens, after the amino acid variation, the lysine sumoylation still happens, but the E3 ligase is changed to another type (expressed as E3^b^ in Fig. 2);(iv) Type IV, the amino acid variations that occur on the adjacent positions of sumoyaltion sites may change the circumstance around the central lysine (K) and further transform the type of PTMs that it should have happened. For example, a lysine which should have be sumoylated might convert into acetylated or ubiquitylated because of the change of amino acids around the center lysine site. Due to the intricate mechanisms of various types PTMs and the unpredictability of transformation between different PTMs, we mainly investigate the first three types of SUMOAMVR and the thorough and comprehensive analysis of Type IV SUMOAMVR will be done in the subsequent work. (More detailed information about the classification method and specific examples could be viewed at: http://bioinfo.ncu.edu.cn/SUMOAMVR_Help.aspx.)

### Identification of Potential SUMOAMVR in Human Variant Proteins

With the sumoylation prediction model, three types of potential SUMOAMVR were identified from 12512 human variant proteins. The statistic data for SUMOAMVRs are shown in Table 2. With the default threshold value, there were a total number of 11632 (the percentage of the whole number of human amino acid variations (68779) which this type (11632) accounts for is 17.13%) potential SUMOAMVRs that was recognized by our prediction tool. With the increase of the threshold value, the total amount of SUMOAMVRs that we could identify decline sharply. However, the higher a threshold value is setting, the more reliable and convincing the prediction result we could obtain. The predicted SUMOAMVRs related to proven sumoylation sites are more likely to be true than that of unproven sumoylation sites. Numerous sumoylation sites in humans are constantly being identified. The priority for further research among predicted SUMOAMVRs could be determined based on the confirmation of the sumoylation sites.

### Analysis of Type I SUMOAMVR

In the process of amino acid mutations, a lysine residue which could be sumoylated may change into other type of amino acid residue to eliminate an original sumoylation site that can be classified as Type I (–). Conversely, a new sumoylation site also can be created once there is a non-sumoylation amino acid residue being replaced by a lysine residue and we defined these as Type I (+).Therefore, the Type I SUMOAMVR would play a significant role in the creation or disruption of sumoylaion sites. We total found 55 type I (−) SUMOAMVRs and 4 type I (+) SUMOAMVRs by matching the locations of the variations and those of lysine sumoylation sites registered in UniProtKB/Swiss-Prot, Phospho-SitePlus, HPRD and a number of other databases as well as the relevant literatures. Of those SUMOAMVRs, 15 of them are known to cause diseases and 9 of them are in involved in the adjustment of various functions of organism and the rest of the remaining SUMOAMVRs still need further investigation, as shown in Table 3. For example, the Lys118Arg amino acid variation of Cyclic AMP-dependent transcription factor ATF-7 (UniProtKB/Swiss-Prot ID, P17544) could abolish the K^181^ sumoylation site resulting in ATF-7 wild-type (ATF7WT) and ATF-7 along with Lys118Arg amino acid variation (ATF7K118R) exhibit significantly different transcriptional and promoter binding activities^42^. Hamard *et al.* has been confirmed that ATF-7 is sumoylated *in vitro* and *in vivo* and sumoylation could affect its intranuclear localization by delaying its entry into the nucleus. Furthermore, SUMO conjugation also inhibits ATF-7 transactivation activity by impairing its association with TAF12 in TFIID and blocking its binding to specific sequences within target promoters. Through a direct measurement of transcription specific to a target gene expression upon the changes in ATF-7 sumoylation, analysis clearly shows that transcription of the E-selectin gene transcription is enhanced in the presence of ATF7K118R whereas the expression level of the control gene (b-actin) is unaffected. Meanwhile, as revealed in localization studies, the ATF7WT is dispersed throughout the nucleoplasm in the majority of the cells (about 90%), while it is restricted to a perinuclear distribution in approximately 10% of the cells. The fact that a non-sumoylatable ATF7K118R never accumulates at the nuclear periphery strongly suggests that the nucleoplasmic ATF-7 corresponds to the non-sumoylated fraction of the protein. In addition, the Lys386Asn amino acid variation of cellular tumor antigen p53 protein (UniProtKB/Swiss-Prot ID, P04637) removes the K^386^ sumoylation site and result in breast cancer (DOID:1612), which is a common malignancy originating from breast epithelial tissue. Mutations at more than one locus can be involved in different families or even in the same case^43^. Also, it was reported that the nucleophosmin protein (UniProtKB/Swiss-Prot ID, P06748) contains a Lys263Arg variation at the K^263^ locus that removes the K^263^ sumoylation site and leads to hematopoietic system disease (DOID:74)^44^. Conversely, the nucleophosmin protein (P06748), involve in the survival of the parasite, has an Arg101Lys variation to create a lysine sumoylation site at K^101^. Moreover, a new sumoyation site can be created in CASP8-associated protein (UniProtKB/Swiss-Prot ID, Q9UKL3) that participate in TNF-alpha-induced activation of NF-kappa-B via a TRAF2-dependent pathway because of the Gln1792Lys variation at the K^1792^ locus^45^. In our forecast results, we discovered that there were 893 (the percentage of the whole number of human amino acid variations (68779) which this type (893) accounts for is 1.30%) potential type I SUMOAMVRs which were identified by our system with the default threshold value. Moreover, with the highest threshold value (0.95), we still discovered 21 high confidence level type I SUMOAMVRs. We randomly chose several examples of type I SUMOAMVRs through the substitution of center amino acid sites (see Supplementary Table S2). But the elimination and creation of these sumoylation sites have not yet been testified. Considering that the presences of amino acid residues could directly affect lysine sumoylation so that they are more likely to be the underlying cause of many diseases which requires further investigation.

### Analysis of Type II SUMOAMVR

**Figure 3:**
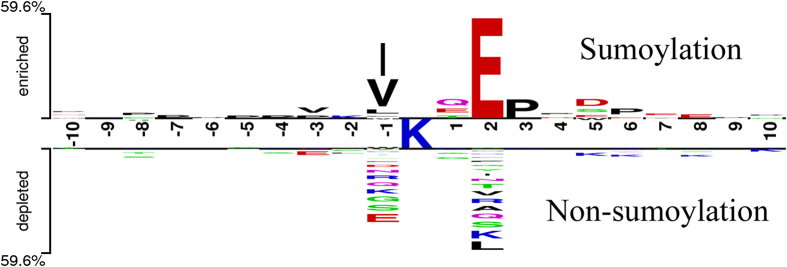
A two-sample logo of the compositional biases around the sumoylation sites compared to the non-sumoylation sites. This logo was prepared using the web server http://www.twosamplelogo.org/ and only residues significantly enriched and depleted surrounding sumoylation sites (t-test, *P*-value <0.05) are shown.

We cannot definitely say that a sumoylation site is changed by a substitution near a sumoylation site because of the complexity of the process of sumoylation. The sumoylation process involves an E1 activating enzyme, an E2 conjugating enzyme, an E3 ligase and a SUMO-protease^46^. It is when the E3 ligase recognized the sumoylation sites that the specific amino acids near the sumoylation sites seem more significant. In this work, we defined the type II SUMOAMVR as the amino acid variations located in the domain (−10 ∼ +10) around the central site to activate (Type II (+)) or inactivate (Type II (−)) the central sumoylation sites. According to results of motif-x, we found that Glutamic acid (E) is the dominant residue at +2 position, and large hydrophobic residue, such as Leucine (L) and Valine (V), prefer to locate at −1 position, which is conforming to previous conclusion that “ΨKxE” might be the consensus motif that E3 ligase could recognize^33^, and also illustrating the reliability of our data. Meanwhile, all of results are in accordance with the result of the Two Sample Logo (*P*-value < 0.05; t test), as shown in Fig. 3, which suggests that fragments between sumoylated and non-sumoylated site have a great difference among the sequence patterns and sumoylation more likely occurs on a lysine in a fragment containing above site characteristics. If these position-specific residues are replaced by other residues, it is highly probable that the adjacent sumoylation sites will be vanished. It is more difficult to observe the examples of type II SUMOAMVRs than those of type I SUMOAMVRs. We only found 5 type II SUMOAMVRs after checking multiple databases and the relevant literatures (Table 4) and three of them are disease related. Ataxin-1 (UniProtKB/Swiss-Prot ID, P54253) overexpressed in transfected cells was found to be sumoylated at multiple lysine residues. Riley *et al.* reported that the sumoylation of Ataxin-1 is dependent on the length of the polyglutamine tract, the ability of ataxin-1 to be phosphorylated at serine^776^, and the integrity of the Nuclear Localization Signal (NLS) of ataxin-1, all of which have a role in the subcellular distribution of ataxin-1^47^. On the one hand, due to ataxin-1 is phosphorylated and SUMO-1 conjugation is regulated by phosphorylation. Therefore, the ataxin-1 construct of Lys^772^ cannot be sumoylated because of Ser776Ala amino acid variation causing the reduction of phosphorylation at serine^776^ and further result in spinocerebellar ataxia autosomal recessive 1 (DOID:1441)^48^, which is a clinically and genetically heterogeneous group of cerebellar disorders. Patients show progressive incoordination of gait and often poor coordination of hands, speech and eye movements, due to degeneration of the cerebellum with variable involvement of the brainstem and spinal cord. On the other hand, to date, the majority of SUMO-1 targets described have been nuclear proteins and, as such, SUMO-1 modification has been suggested to be a predominantly nuclear process. Ataxin-1 has two possible nuclear localization signals, Lys^16^ and Lys^772^, the latter of which has turned out to be a functional NLS. Compared to the majority of substrates reported to date are sumoylated at a single lysine residue, Ataxin-1 is sumoylated on at least five residues, Ataxin-1 with a mutated NLS showed a dramatic decrease in its ability to be sumoylated^47^. In addition, the phosphorylation of the motif ΨKXEXXpSP (Ψ is a hydrophobic residue, X is any amino acid, pS is a phosphorylatable serine) at S^303^ of HSF1 (UniProtKB/Swiss-Prot ID, Q00613), a heat shock transcription factor, enhances the adjacent lysine sumoylation at K^298,^^49^. But the occurrence of variation of the Ser303Ala at the S^303^ removes the K^298^ sumoylation site. Also, it was reported that the Prelamin-A/C protein (UniProtKB/Swiss-Prot ID, P02545) harbors a Gln203Gly mutation in the rod domain which is associated with aberrant localization with decreased nuclear rim staining and formation of intranuclear foci causing dilated cardiomyopathy and conduction-system disease without skeletal myopathy. When the glutamic acid (E) was replaced by glycine (G), the sumoylation site at the Lys^201^ locus was decreased^50^. In addition to the above cases, we also found Ile92Ala of the ubiquitin carboxyl-terminal hydrolase 25 protein (UniProtKB/Swiss-Prot ID, Q9UHP3) and Lys6Arg of the U4/U6.U5 tri-snRNP-associated protein 2 (UniProtKB/Swiss-Prot ID, Q53GS9) could repeal their neighboring sumoylation sites^51^,^52^. Form our prediction results, we total detected 2738 (the percentage of the whole number of human amino acid variations (68779) which this type (2738) accounts for is 3.98%) potential type II SUMOAMVRs from 12512 protein sequences with the default threshold value. We select several examples of potential type II SUMOAMVRs that abolish and activate the sumoylation sites through the substitution of adjacent amino acid residues (see Supplementary Table S3). But the abolishment and activation of these sumoylation sites have not yet been confirmed. The presences of specific amino acid residues do not directly affect lysine sumoylation by all ligase E3, but the sequences near the sumoylation sites must be considered.

### Analysis of Type III SUMOAMVR

Type III SUMOAMVR are of a certain kind variations that change only the type of enzymes involved rather than affecting the sumoylation site itself. Up to now, three different types of SUMO ligase E3 has been identified including PIAS protein family (protein inhibitors of activated STAT), NucleoporinRanBP2/Nup358 (Ran binding protein-2) and Pc (human polycomb group protein), but the enzymes activities, functions and exact substrates of a large proportion of them still remained to be experimentally identified^53^. It is when the E3 ligase recognized the sumoylation sites that marking the beginning of sumoylation. Based on a widely accepted hypothesis that similar enzymes could recognize similar patterns^27^, it is meriting our consideration that an amino acid mutation on a substrate could cause the impact on the corresponding enzyme. For example, Zinc finger protein 131 (ZNF131) is a target for SUMO modification and as a substrate for the SUMO E3 ligase human polycomb protein 2. SUMO modification potentiates the negative effect of ZNF131 on estrogen signaling and consequently attenuates estrogen-induced cell growth in a breast cancer cell line. But the variation of the Thr497Ala in E3 SUMO-protein ligase CBX4 results in small extent decrease in ZNF131 sumoylation^54^. In addition, Phe385Ala at F^385^ in cellular tumor antigen p53 (UniProtKB/Swiss-Prot ID, P04637) could reduce the level of K^368^ SUMO1 conjugation^55^ (Table 5). We total detected 8001 (the percentage of the whole number of human amino acid variations (68779) which this type (8001) accounts for is 11.63%) potential type III SUMOAMVRs from 12512 protein sequences with the default threshold value, as shown in Table 2. In our prediction results, we have counted that the number of type III SUMOAMVRs (8001) accounted for 68.78%of the whole of the detected SUMOAMVRs (11632), indicating that the type III SUMOAMVR might play predominant roles in affecting protein sumoylation states to rewire signaling pathways rather than activating or removing an sumoylation site directly. Several examples of potential type III SUMOAMVRs were listed in Table S4 (see Supplementary) and our prediction results could also provide a useful resource for further considerations of experimental medicinal.

### Statistical Analysis of Different Types of SUMOAMVR

We have collected all information about human polymorphic and diseases’ mutations that are consist of three aspects of SNP, diseases and unclassified variations. In this part, disease-associated and polymorphic mutations were investigated on their affection of sumoylation of protein with the default threshold value. Based on the results, we found that 4.83% of mutations of disease related and 9.58% of polymorphic mutations could result in the SUMOAMVR, as shown in Table 6. Therefore we have reasons to believe that the vast majority of SUMOAMVRs are caused by polymorphic mutations except the type I (+) SUMOAMVR (*P* = 0.09), which means that the amino acid mutations that directly add the sumoylation sites are more likely to cause the occurrence and development of disease. Taken together, mutations associated with the polymorphism accounted for a large proportion of all SUMOAMVRs, especially in Type II (−) and Type III SUMOAMVRs. However, with the development of medical science and technology, and further understanding of the pathogenesis of the disease, more and more SUMOAMVRs associated with the disease will be found.

### Functional Effects of Sumoylation in the Pathogenesis of the Human Diseases

**Figure 4:**
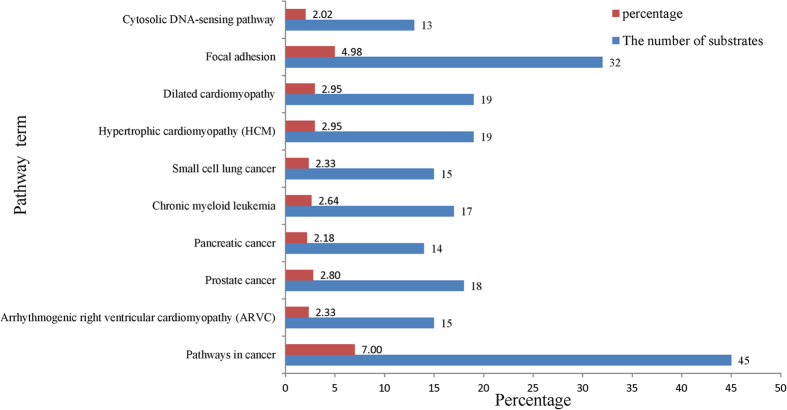
The data statistics of pathway terms for disease-related sumoylation substrates on the background of normal sumoylation substrates. The blue pillar represent the number of substrates of different pathway terms and the red pillar represent the percentage of the number of substrates of different pathway terms in all substrates. Statistical significance (*P*-value) gradually increased from top to bottom.

In order to further investigate whether the amino acid substitutions that occur in the vicinity of the sumoylation sites impact the state of sumoylation and thus imperceptibly have an influence on the expression of many bodily functions of the human as well as the possible impact of the disease process, we have probed the state of the expression of functional elements with our data such as pathways and gene ontology (GO). Firstly, we used DAVID program^56^,^57^ to analyze the pathway to further explore functional aspects of disease-related sumoylation substrates. The top 10 statistically significant results (*P* < 0.05) were listed in Fig. 4. From the results, we could detect that a large portion of disease-related sumoylation substrates were involved in cancer pathways including pathways in cancer, pancreatic cancer, prostate cancer, non-small cell lung cancer. In statistical results obtained, the number of genes involved in the term of "Pathways in cancer" is not simply count up the number of all of other 14 specific cancers such as "pancreatic cancer" and "prostate cancer", etc. But with all of other 14 specific cancers are listed as an individual subtype alone, due to genes known to be functionally altered in each of 14 specific types of cancers are highlighted on their pathway maps only, some of such genes are not highlighted on "Pathways in cancer". The reason is that such oncogenes and tumor suppressor genes are different depending on cancer type^58^. The occurrence of these cancers is highly related to small ubiquitin-related modifier (SUMO) conjugation or sumoylation. By masking or adding interaction surfaces or by inducing conformational changes, the sumo protein governs protein-protein and protein-DNA interactions and in consequence can lead to changes in protein localization, activity or stability^5^. There are numerous lines of evidence point to the role for SUMO in carcinogenesis. Many activities of important tumor suppressors and oncoproteins are regulated by sumo including PML, WRN, BLM, c-JUN, c-FOS, TP53 and MDM2^59^,^60^,^61^,^62^,^63^ and of several nuclear hormone receptors including estrogen receptor alpha (ERa), progesterone receptor (PR) and AR^64^, which play a central role in the development of hormone drive breast tumors^65^, are modified by SUMO in a ligand-dependent manner. For example, mutations of ERa that prevent SUMO modification impair the transcriptional activity of the receptor^66^. In addition, various components of the SUMO-conjugating machinery are upregulated in several malignancies: UBC9 in melanomas, ovarian cancer and lung adenocarcinomas^67^,^68^,^69^, the SUMO isopeptidase SENP1 in prostate cancer^70^ and PIAS3 in breast, lung, prostate, colorectal and brain tumors^71^, indicating that through affecting the state of SUMO, genetic variability might disturb the normal sumoylation of protein and further have an effect on the occurrence of many diseases. The other parts of the result were involved in Cytosolic NDA-sensing signaling pathway, Hypertrophic cardiomyopathy, etc., which also participate in adjusting the process of mechanisms of various functions of human body. Furthermore, dynamic sumoylation hypothetically orchestrates a wide range of cellular processes containing matrix metabolism, inflammation, survival, senescence and autophagy in cell populations. Based on the result of the enrichment of substrates, we also found a number of substrates involved in these processes. We discovered 12 substrates that are involved in "VEGF signaling pathway"(map04370), which may participate in mediating processes of cell survival. There is now much evidence that the binding of VEGF to VEGFR-2 leads to a cascade of different signaling pathways, resulting in the up-regulation of genes involved in mediating the proliferation and migration of endothelial cells and promoting their survival and vascular permeability. For example, the activation of the phosphatidylinositol 3' -kinase (PI3K)-Akt pathway could leads to increased endothelial-cell survival^72^. In addition, there are 28 substrates participate in "MAPK signaling pathway"(map04010) which may adjust processes of inflammation in cell. The mitogen-activated protein kinase (MAPK) cascade is a highly conserved module that is involved in various cellular functions. Some MAPKKKs may activate ERK1/2 in response to pro-inflammatory stimuli^73^. Moreover, Menendez *et al.* have reported that mTOR could regulate senescence and autophagy during reprogramming of somatic cells to pluripotency^74^. We also found 8 substrates involved in "mTOR signaling pathway" (map04150) which may have affection on senescence and autophagy in cell populations. The detailed data of pathway enrichment are listed in Table S5. (see Supplementary).

The analysis of disease-related protein of sumoylation and normal protein of sumoylation from databases provided us an opportunity to profoundly understand the impact of sumoylation in the pathogenesis of the human disease as well as broad effects variations might have. In this part, we statistically analyzed the enriched biological processes, molecular functions and cellular components with the gene ontology (GO) annotations and compared the differentiated GO terms with Fisher exact test (Two-sided category) for disease-related protein of sumoylation and normal protein of sumoylation. The top 5 statistically over-represented terms of these three criteria are shown in Table 7, of which we could detect that regulation of transcription (GO:0006357, GO:0045941), regulation of gene expression (GO:0010628), etc., regulatory mechanisms in biologic processes are the most apparent differentiated GO terms and they all present over-represented in disease-related protein. For molecular functions, the DNA binding functions such as sequence-specific DNA binding (GO: 0043565), structure-specific DNA binding (GO: 0043566) and double-stranded binding (GO: 0003690) and the transcriptional activity (GO: 0016563, GO: 0003702) are the most apparent differentiated GO terms. According to the analysis cellular component, the diseased sumoylated substrates are more likely to localized in intracellular organelle and membrane (such as GO: 0070013, GO: 0043233 and GO: 0031974). The above analysis showed that there might have significant differences in biological processes, molecular functions and cellular components for diseased and normal sumoylated substrates indicating that the occurrence of many diseases might be due to changes of the state of normal sumoylation, and also offer more instructive clues to identify the mechanisms of abnormal sumoylation related diseases, which would be helpful to design the related drugs to prevent or treat possible diseases.

### Computation Programs Construction and Web Server

It is our foremost purpose to build an open platform which could provide comprehensive and more in-depth analysis of sumoylation of human proteins in silicon prediction. Based on the Asp.net(C#) and Matlab, a web platform service was constructed in an easy-to-use manner and is available for the general public at: http://bioinfo.ncu.edu.cn/SUMOAMVR_Home.aspx. Bug fixing and minor changes of sumoylation prediction model will be done. The improved sumoylation prediction model will be constructed when the new sumoylation sites data become available.

## Discussion

As the rapid development of sequencing techniques, a large number of genetic variations are emerging. It has been become extremely urgent to select the meaningful variations among the endless numbers of newly identified polymorphisms for most scientific researchers. Protein function has been observed to rely on select essential sites instead of requiring all sites to be indispensable^75^. Sumoylation represents an important class of PTMs in which a SUMO protein is covalently attached to a protein, which has the capacity to regulate multiple biochemical properties of the protein target. In consideration of the crucial role that SUMO conjugation act in orchestrates a variety of cellular processes, from the aspects of the effect of the genetic mutations on the process of sumoylation in protein might be a very valuable way to explore underlying connection of human genetic polymorphisms and variations with plenty of vital biological functions. In this respect, our system can really provide more instructive guidance to identify the mechanisms of genetic diseases. For example, Wen *et al.* 2014, demonstrated that K^6^, K^16^, K^29^, K^51^, and K^73^ were the sumoylation sites of USP39. Mutation of above lysine residues could eliminate these original sumoylation sites of USP39 and further promoted the proliferation-enhancing effect of USP39 on prostate cancer cells^76^. With our system, the predicted results are identical with experimental verification as the literature demonstrated that K^29^>, K^51^, and K^73^, and, to a lesser extent, K^6^ are the SUMO acceptor sites of USP39 as shown in Table S6 (see Supplementary). While lysines which could be sumoylated were mutated into arginines, these sumoylation sites were abolished as well.

In the past decade we have witnessed rapid progress in the functional dissection of protein sumoylation^4^. To fully decipher the molecular mechanisms of sumoylation-related biological processes, an initial but crucial step is the identification of sumoylated substrates and the corresponding sumoylated sites. With mass spectrometry (MS) approaches, several large-scale experiments of sumoylated substrates have been carried out^77^,^78^,^79^. However, the complexities of sumoylation mechanism cannot be perfectly solved by experimental approaches, especially those with dozens of potential consensus and non-consensus sumoylation sites. Mutational analysis would be labor-intensive and time-consuming. Parallel to the experimental identification of sumoylated site, computational prediction of sumoylation sites in silico might represent a promising method for its accuracy, convenience and speed, especially models for performing large-scale predictions,. In this work, since we have considered the different aspects of the amino acids properties when we build the model so that our system can provide a more comprehensive prediction performance comparing with existing methods owing to avoid bias of the prediction results because of the single manner of feature extraction of many existing methods. Compared to the many other tools, it is worth mentioning that the formula of the SumoPred could attain superior performance. Although there is a slight extent of probability that human SNPs could result in amino acid variations, the impact of these variations is severe and profound^27^. In 2010, Li *et al.* have investigated the amino acid variations associated with the 15 different PTMs and found that about 4.5% of amino acid variations may affect protein function through disruption of post-translational modifications^25^. Changes in sumoylation sites cause various diseases by numerous mechanisms. Therefore, once the state of sumo target substrate was changed because of the amino acid variations, diverse cellular processes involved in sumoylation, including nuclear transport, cell cycle control and maintenance of genome integrity, would also change accordingly leading to changes in protein localization, activity or stability. Some proven mechanisms of the SUMOAMVRs have shown in introduction and concrete analysis above and Tables 3, 4, 5 are related to changes in the protein’s affinity for DNA, abolishing enzymatic activity and slow down of ligand-dependent nuclear translocation. But considering the significance function roles of sumoylaton *in vivo*, there must be many mechanisms which SUMOAMVR can cause specific diseases and these must be identified.

Meanwhile, an increasing number of studies have indicated that different types of PTMs synergistically orchestrate specific biological processes by crosstalk. However, the preference of the crosstalk among different PTMs and the evolutionary constraint on the PTM crosstalk need further dissections. From the aspects of direction of the type IV SUMOAMVR might be a very valuable way to explore underlying connection of the crosstalk among different PTMs. The amino acid at the same positions of a fragment could occur several lysine PTMs including sumoylation, acetylation and ubiquitylation, etc. Therefore, the amino acid variations that occur on the adjacent positions of sumoyaltion sites may change the circumstance around the center lysine (K) and further transform the type of PTMs or due to variations leading to the occurrence of a kind of modified on the adjacent positions thus impact the type of PTM of central site that it should have happened. A recently computational analysis suggested that a considerable proportion of acetylated lysine might influence the PTMs such as, methylation and ubiquitination of adjacent sites^80^, Moreover, one PTM can regulate another PTM by modifying its cognate enzymes and vice versa in a trans-regulatory mode^81^,^82^,^83^. For example, the E3 ubiquitin ligase complex of Rictor/Cullin-1/Rbx1 ubiquitinates an AGC kinase of SGK1 and promote its degradation, whereas the T^1135^ of Rictor can be phosphorylated by multiple AGC kinases including SGK1, and such a phosphorylation disrupts the interaction of Rictor and Cullin-1 to inhibit the ubiquitination of SGK1^82^. Due to similar theory above, a lysine which should have be sumoylated might convert into acetylated or ubiquitylated because of the change of amino acids around the central lysine sites. And comprehensive analysis of Type IV SUMOAMVR will be done in the subsequent work.

When personalized medicine is the next frontier for scientists, industry and the general population, it is becoming more significance to exploit computational approaches that can lead to a better understanding of the etiology of disease. Integration of genetic and molecular information is a sensible step in this direction because it provides a structural and functional perspective to the human variation data. Our approach can not only be adopted in pathophysiological diagnosis researches of mutations, but also play a critical role in the selection of polymorphisms of clinical and phenotypical importance. Up to now, many articles have a detailed report about variations, did not mention that the variations could be related to changes in lysine sumoylated sites. This could be largely attributable to the shortage of a specific research about the relationship between mutations with lysine sumoylation sites, or the lack of a general understanding of the association between lysine sumoylation and mutation. Accordingly, many nonsense point mutations whose functional mechanisms are unknown can be reconsidered in terms of SUMOAMVR. Furthermore, if some mutations are predicted to be SUMOAMVRs with our system, further research will clarify the cause of the associated disease or protein function. Our study can be adopted to select the meaningful variations among the endless numbers of newly identified polymorphisms. With the rapid progress of sequencing technologies and the appearance of new methods, a large number of genetic variations are emerging along with this advance of biotechnology. At present, it is possible to make comparison of the whole genomes of individuals and a comparison of SUMOAMVR between individuals or between species can be utilized before amino acid variations or genetic variability are compared in whole genomes. A reverse genetic approach for unknown protein functions or phenotypic variations is possible with proven SUMOAMVR. The screening and prediction of SUMOAMVR can be a starting point for further research.

## Methodology

### Data collection and preprocessing

The data sets of lysine sumoylated sites sequences were collected from several public databases, including UniProtKB/Swiss-Prot(http://www.uniprot.org), PhosphoSitePlus (http://www.phosphosite.org)^31^ and HPRD (http://hprd.org/)databases^32^. In total, we collected 498 experimental sumoylated proteins from those databases and these data sets contained 752 unique sumoylation sites and 19202 non-sumoylation sites by wiping off homologous fragments with a sensitive cutoff of 0.3 using the CD-HIT^84^. In our system, a local sliding window size of a maximum number of 10 residues flanking each lysine was chosen because the fragments of this size could cover the 10-amino acid NDSM region located downstream from the consensus motif-ΨKxE (where Ψ is a large aliphatic branched hydrophobic amino acid and x is any amino acid)^85^ Then, the positive sets were composed of 752 sumoylated fragments. It would be difficult to prove definitively that a lysine residue is not sumoylated under any conditions. To circumvent this problem of choosing the negative sets (non-sumoylated sites), we made the assumption^86^ that the non-sumoylated site is lysine residue that has not been marked by any sumoylation information on the same proteins, the rationale of which is that the resulting negative samples are more likely to be non-sumoylation sites than those obtained by random as these proteins were experimentally investigated. Although not all of these non-sumoylated sites are necessarily true negatives, it is reasonable to expect that a large majority of them are.

### Feature extraction

The extracted features were classified into three major categories: sequence-derived features, structure features, and evolutionary information of amino acids. In keeping with earlier studies and also covering the 10-amino acid NDSM region located downstream from the consensus motif-ΨKxE (where Ψ is a large aliphatic branched hydrophobic amino acid and x is any amino acid)^85^ a local sliding window size of a maximum number of 10 residues flanking each lysine was employed to extract the multiple features of each candidate residue. In total, 401 features from different feature types were extracted (see Supplementary Table S7).

### Feature optimization and selection

These above features that we adopt to develop our system are consist of many dimensions, some of which may not be valuable to the prediction of sumoylation sites and they could be also redundant with each other because of the specificity of the sumoylated substrates. Therefore, we introduced a feature selection method known as F-select^34^ to remove the irrelevant and redundancy feature vectors. The selection method was performed using the 10-fold cross validation strategy for each of the ten training sets as follows. First, averaged F-score values of the each ten training sets were calculated with the purpose of ranking the features vector. We used a wrapper-based feature selection with the forward best first search. More specifically, for a given list of feature F = [*f*i where i = 1, 2… n] sorted in the descending order by their average F-score value and an empty list R that store the selected features. We add the top-ranked feature from F to R and run SVM using feature set R in the cross validation strategy. If the addition of the top ranked feature improves the average accuracy value over the ten test folds, then this features vector is retained in R; otherwise it will be removed. We repeat that until F is empty. Finally, the SVM classifier is trained to distinguish sumoylation and non-sumoylation sites on the selected feature set. The F-score of *i*th feature is defined as;


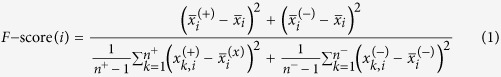


Where 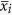, 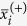 and 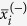 are the average value of the *i*th feature in whole, positive and negative data sets, respectively. *n*^+^ denotes the number of positive data, *n*^−^ denotes the number of negative data, 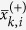 denotes the *i*th feature of the *k*th positive instance, and 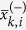 denotes the *i*th feature of the *k*th negative instance.

Based on biological characteristics of sequence, structural and evolutionary information surrounding sumoylation sites and non-sumoylation sites, this study have assessed multiple features and eventually adopted 5 encodings schemes including physicochemical property of amino acid, CKSAAP encoding, AASA feature, secondary structure of amino acid and PSSM and further optimized by F-score as the input vector to train our model.

The true positive rate and the false positive rate are calculated to draw the receiver operating characteristic (ROC) curve and we use the area under the curve (AUC) to quantify the predictive quality. Figure S1 displays ROC curves of different encoding before and after optimization based on the measurement of F-score using a 10-fold cross-validation strategy (there is no obvious difference before and after optimization regarding physicochemical property of the amino acid, the curve did not show here). Apart from physicochemical property, we found that all the AUC of the optimized features have a relatively obvious improvement (see Supplementary Figure S1) and AUC value of fusion features is also increased from 0.8519 to 0.9038 (see Supplementary Figure S2) suggesting these features set after optimized are more capable of distinguishing sumoylation sites and non- sumoylation sites.

### Selection of balanced negative set and cross-validation

Since the number of negative samples is far more than the positive samples, to avoid such overweighting by this extreme imbalance, we randomly choose the same number of negative samples as the number of positive samples in this study. Here, 10 sets of negative samples were constructed. For each set of negative samples, a 10-fold cross validation test was executed by randomly dividing positive samples and negative samples into training and test sets in a 9:1 ratio. The 10-fold cross-validation test was performed 10 times.

### Selecting the optimal parameter for the SVM

In our work, we adopt LIBSVM package to train data sets (version 3.1), which can be freely downloaded from http://www.Csie.Ntu.Edu.Tw/~cjlin/libsvm/. And a radial basis function (RBF) was selected as the kernel function. Meanwhile, based on the training sets, two parameters including the penalty parameter C and the kernel width parameter γ were tuned in order to find the best parameters. Finally, the parameters C = 8 and γ = 0.001953, which could resulted in the best performance (i.e. the average Ac over all the cross-validation is the highest), were considered as the optimal SVM parameters of the our model.

### Prediction model evaluation

In this paper, four measurements, the accuracy (Ac), sensitivity (Sn), specificity (Sp) and the mattews correlation coefficient (MCC), which are commonly used in other studies, were applied to assess the prediction performance. The definitions are as follows:


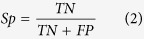



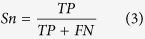



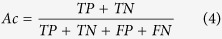



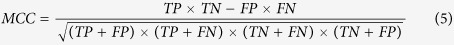


These parameters were defined in terms of the true positive (TP), false negative (FN), true negative (TN), and false positive (FP).

In the formula, the accuracy denotes the percent of correct prediction in all the positive and negative sets. The sensitivity (the true positive rate) and the specificity (the true negative rate) represent the percentage of the positive set and the negative set that were correctly predicted, respectively. Among these values, the MCC value is the most important measurement when considering the highly imbalanced training dataset used here, and a higher value always indicates a better prediction performance.

### SVM scores for the predicted results

For the scores of the predicted results in our system, the probability estimates of the LIBSVM package was adopted. The probability estimates are calculated using the decision values of the SVMs and have values ranging from 0 to 1. The values of true positive sites converge to 1, while those of true negative sites get close to 0. In order to reduce the number of false positives, we based the options on specificity to evaluate the prediction model. The default probability estimate of LIBSVM for classification was 0.5, and the specificity of prediction increased if the probability estimate for the classification approached a value of 1.

### Statistical Analysis

In the analysis of article, two strategies were introduced to estimate statistical confidence of the results. First, t-test^87^ as adopted while we develop the lysine sumoylation prediction model, of which a *P*-value < 0.05 is expected to be statistically significant. Furthermore, we used the Fisher's exact test with the 2 × 2 contingency table method to evaluate statistical confidence of the observed trends for all the predicted SUMOAMVRs^88^. In other words, Fisher's exact test was applied to determine whether a certain type of SUMOAMVR is enriched or depleted in disease-associated single amino acid mutations and SNP in protein sumoylation.

### Sequence Logos

We total obtained 752 sumoylated sequences after dealing with several databases, which were presented as fragments of 21 residues with the residue K in the central position. Then, we utilized a tool named motif-x^89^ to analyze the lysine sumoylation data against the background set of all non-sumoylation sequence fragments. Finally, according to the results of analysis, we discovered 7 significant motifs (see Supplementary Figure S3, Table S8), all of which are conforming to the result of the Two Sample Logo^90^ (*P*-value < 0.05; t-test), as shown in Fig. 3.

## Additional Information

**How to cite this article**: Xu, H.-D. *et al.* Systematic Analysis of the Genetic Variability That Impacts SUMO Conjugation and Their Involvement in Human Diseases. *Sci. Rep.* **5**, 10900; doi: 10.1038/srep10900 (2015).

## Supplementary Material

Supplementary Information

## Figures and Tables

**Table 1:** Comparison of predictive performance of SumoPred with other predictors.

method	stringency	the performance of prediction			
		Ac (%)	Sn (%)	Sp (%)	MCC (%)
GPS-SUMO	Low	66.67%	78.79%	54.55%	34.36%
	Medium	77.23%	72.73%	81.82%	54.73%
	High	72.73%	51.52%	**93.94%**	50.20%
SeeSUMO	Low	69.70%	**90.91%**	48.48%	43.50%
	Medium	63.14%	75.76%	51.52%	28.11%
	High	51.53%	9.09%	**93.94%**	5.73%
SumoPred	—	**84.85%**	87.88%	81.82%	**69.85%**

^a^GPS-SUMO, SeeSUMO and SumoPred were tested using an entirely independent dataset ^b^The highest values for each threshold are indicated in bold. Abbreviations: Ac, Accuracy; Sn, Sensitivity; Sp, Specificity; MCC, Matthews correlation coefficient.

**Table 2:** The data statistics for three types of SUMOAMVR detection in different specificity options^a^.

Specificity	Type1		All	Type11		All	Type111
	Type1(+)	Type1(−)		Type11(+)	Type11(−)		
Default	512	381	893	1364	1374	2738	8001
70%	235	176	411	645	689	1334	3552
80%	135	102	237	413	431	844	2053
90%	34	45	79	194	203	397	760
95%	9	12	21	75	88	163	220

^a^We used SumoPred to predict sumoylation sites after taking into account of the amino acid variations in human proteins.

**Table 3:** The examples of type I SUMOAMVR, which include the change of an amino acid with lysine residue or vice versa to create a potential new (Type I (+)) or remove an original lysine sumoylation site (Type I (−)).

Gene name (UniProtKB ID)	Variation site^a^	Sumo- ylation site	Local peptide sequence^b^	Modify Source^c^	Variation Source^d^	Status^e^	MIM^f^
Type I (−) SUMOAMVRs							
TP53 (P04637)	Lys386Asn	K386	STSRHKKLMF**K**T EGPDSD	Phosphositeplus	IntOGen	DOID:1612-breast cancer	114480
NPM1 (P06748)	Lys263Arg	K263	ASIEKGGSLP**K**V EAKFINYVK	Phosphositeplus	ICGC; IntOGen	DOID:9119-acute myeloid leukemia; DOID:74-hematopoietic system disease	601626
CBS (P35520)	Lys211Arg	K211	ESHVGVAWRL**K**N EIPNSHILD	Phosphositeplus	dbSNP	—	—
NR1H3 (Q13133)	Lys434Asn	K434	QVFALRLQDK**K**L PPLLSEIWD	Phosphositeplus	dbSNP	—	—
ZNF221 (Q9UK13)	Lys423Ile	K423	QQVHSGQKSF**K**C EECGKGFYT	Phosphositeplus	TCGA	—	—
FOSL2 (P15408)	Lys222Asn	K222	GGGSVGAVVV**K**Q EPLEEDSPS	Phosphositeplus	TCGA	—	—
NR1H3 (Q13133)	Lys328Gln	K328	DFSYNREDFA**K**A GLQVEFINP	Phosphositeplus	dbSNP	—	—
INO80B (Q9C086)	Lys168Asn	K168	KGELDDNGDL**K**K EINERLLTA	HPRD9.0	nextProt	—	—
HIF1A (Q16665)	Lys477T	K477	DPALNQEVAL**K**L EPNPESLEL	Phosphositeplus	TCGA	—	—
PML;MYL (P29590)	Lys487Asn	K487	RKCSQTQCPR**K**V IKMESEEGK	Phosphositeplus	ICGC	—	—
SALL1 (Q9NSC2)	Lys1086Asn	K1086	IPANSLSSLI**K**T EVNGFVHVS	HPRD9.0	TCGA	—	—
IRF2 (P14316)	Lys166Ile	K166	PEYAVLTSTI**K**N EVDSTVNII	UniProtKB	dbSNP	—	—
RSBN1 (Q5VWQ0)	Lys313Asn	K313	GLNKESFRYL**K**D EQLCRLNLG	HPRD9.0	dbSNP	—	—
SREBF1 (P36956)	Lys123Gln	K123	MPAFSPGPGI**K**E ESVPLSILQ	HPRD9.0	dbSNP	—	—
ARID5B (Q14865)	Lys629Asn	K629	MADYIANCTV**K**V DQLGSDDIH	HPRD9.0	ICGC;TCGA; COSMIC; IntOGen	DOID:2871-endometrial carcinoma; DOID:9460-uterinecorpus cancer	608089
ACTB (P60709)	Lys68Gln	K68	AQSKRGILTL**K**Y PIEHGIVTN	Phosphositeplus	dbSNP	—	—
SCIN (Q9Y6U3)	Lys299Gln	K299	AAKQIFVWKG**K**D ANPQERKAA	Phosphositeplus	COSMIC	DOID:234-colon adenocarcinoma	—
ZBTB1 (Q9Y2K1)	Lys328Gln	K328	RAAERKRIII**K**M EPEDIPTDE	Phosphositeplus	nextProt	DOID:2526-prostate adenocarcinoma	176807
VHL (P40337)	Lys171Asn	K171	RCLQVVRSLV**K**P ENYRRLDIV	Phosphositeplus	nextProt; COSMIC	DOID:4465- papillary renal cell carcinoma	605074
PIAS4 (Q8N2W9)	Lys128Met	K128	GLGRLPAKTL**K**P EVRLVKLPF	UniProtKB	nextProt; dbSNP	—	—
HIST1H4A (P62805)	Lys13Ile	K13	GRGKGGKGLG**K**G GAKRHRKVL	Phosphositeplus	nextProt; COSMIC; IntOGen	DOID:1749-squamous cell carcinoma; DOID:8557-oropharynx cancer	—
HIST1H4A (P62805)	Lys13Asn	K13	GRGKGGKGLG**K**G GAKRHRKVL	Phosphositeplus	TCGA; IntOGen	DOID:8557-oropharynx cancer	—
SNCA (P37840)	Lys96Arg	K96	GSIAAATGFV**K**K DQLGKNEEG	Phosphositeplus	ICGC;TCGA	DOID:234-colon adenocarcinoma	—
IGF1R (P08069)	Lys1150Arg	K1150	NCMVAEDFTV**K**I GDFGMTRDI	Phosphositeplus	TCGA; COSMIC	—	—
BLM (P54132)	Lys347Asn	K347	TSKDLLSKPE**K**M SMQELNPET	Phosphositeplus	NCI-60panel	—	—
HDAC1 (Q13547)	Lys476Gln	K476	KEEKPEAKGV**K**E FEVKLA	Phosphositeplus	NCI-60panel	—	—
PDE4D (Q08499)	Lys387Asn	K387	TNSSIPRFGV**K**T EQEDVLAKE	UniProtKB	TCGA	—	—
UBA2 (Q9UBT2)	Lys257Asn	K257	TGYDPVKLFT**K**L FKDDIRYLL	Phosphositeplus	TCGA	—	—
BLM (P54132)	Lys344Asn	K344	VLSTSKDLLS**K**P EKMSMQELN	Phosphositeplus	dbSNP	—	—
MAPKAPK2(P49137)	Lys353Arg	K353	KEDKERWEDV**K**E EMTSALATM	Phosphositeplus	nextProt; dbSNP	—	—
CHD7 (Q9P2D1)	Lys1196Asn	K1196	MLRRLKEDVE**K**N LAPKEETII	Phosphositeplus	ICGC;TCGA	DOID:2871-endometrial carcinoma	608089
XRCC4 (Q13426)	Lys210Arg	K210	NAAQEREKDI**K**Q EGETAICSE	Phosphositeplus	dbSNP	—	—
USP25 (Q9UHP3)	Lys141Thr	K141	RVLEASIAEN**K**A CLKRTPTEV	Phosphositeplus	dbSNP	—	—
RLF (Q13129)	Lys1561Thr	K1561	CMVQGCLSVV**K**L ESSIVRHYK	HPRD9.0	TCGA	—	—
TBX22 (Q9Y458)	Lys63Asn	K63	GKSEPLEKQP**K**T EPSTSASSG	Phosphositeplus	nextProt; COSMIC; IntOGen	DOID:8557-oropharynx cancer	—
SUMO1 (P63165)	Lys16Asn	K16	AKPSTEDLGD**K**K EGEYIKLKV	Phosphositeplus	TCGA; COSMIC	—	—
KCNIP3 (Q9Y2W7)	Lys90Gln	K90	DQLQAQTKFT**K**K ELQSLYRGF	Phosphositeplus	TCGA	—	—
HIF1A (Q16665)	Lys391Arg	K391	EDTSSLFDKL**K**K EPDALTLLA	Phosphositeplus	TCGA	—	—
RANBP2 (P49792)	Lys2725Gln	K2725	EKKPTVEEKA**K**A DTLKLPPTF	Phosphositeplus	TCGA	—	—
IRF2 (P14316)	Lys137Gln	K137	TEKEDKVKHI**K**Q EPVESSLGL	UniProtKB	COSMIC	DOID:684-hepatocellular carcinoma	114550
POLD3 (Q15054)	Lys433Gln	K433	SVHRPPAMTV**K**K EPREERKGP	Phosphositeplus	ICGC;TCGA; COSMIC; IntOGen	DOID:1324-lung cancer; DOID:3907-lung squamous cell carcinoma	211980 608935 612593 614210
ZNF462 (Q96JM2)	Lys2482Asn	K2482	DEAIGIDFSL**K**N ETVAICVVT	HPRD9.0	TCGA	—	—
MDM4 (O15151)	Lys254Asn	K254	SVSEQLGVGI**K**V EAADTEQTS	Phosphositeplus	dbSNP	—	—
APP;A4 (P05067)	Lys670Asn	K670	NIKTEEISEV**K**M DAEFRHDSG	Phosphositeplus	nextProt	—	—
UBA2 (Q9UBT2)	Lys623Asn	K623	KLDEKENLSA**K**R SRIEQKEEL	Phosphositeplus	ICGC;TCGA	DOID:2871-endometrial carcinoma	608089
SUMO1 (P63165)	Lys7Gln	K7	MSDQEA**K**P STEDLGDKK	Phosphositeplus	TCGA; IntOGen	DOID:1324-lung cancer	211980 608935 612593
HSF1 (Q00613)	Lys298Asn	K298	PLSSSPLVRV**K**E EPPSPPQSP	UniProtKB	dbSNP	—	—
USP25 (Q9UHP3)	Lys99Gln	K99	TNVIDLTGDD**K**DDLQRAIALS	UniProtKB	dbSNP	—	—
NF2 (P35240)	Lys76Arg	K76	YTIKDTVAWL**K**M DKKVLDHDV	Experimental verification	—	Disrupts merlin cortical cytoskeleton residency and attenuates its stability	—
AGO2 (Q9UKV8)	Lys402Arg	K402	PYVREFGIMV**K**D EMTDVTGRV	Experimental verification	—	Regulate sits stability	—
RARA (P10276)	Lys399Arg	K399	AKGAERVITL**K**M EIPGSMPPL	UniProtKB	dbSNP	Regulates its subcellular localization and transcriptional activity	—
ATF7 (P17544)	Lys118Arg	K118	SLPSTPDIKI**K**E EEPVEVDSS	UniProtKB	—	Impact transcriptional and promoter binding activities	—
RARA (P10276)	Lys166Arg	K166	ESVRNDRNKK**K**K EVPKPECSE	UniProtKB	dbSNP	Regulates its subcellular localization and transcriptional activity	—
RARA (P10276)	Lys171Arg	K171	DRNKKKKEVP**K**P ECSESYTLT	UniProtKB	dbSNP	Regulates its subcellular localization and transcriptional activity	—
ZIC3 (O60481)	Lys248Arg	K248	AFFRYMRQPI**K**Q ELSCKWIDE	Experimental verification	—	Regulates nuclear localization and function of zinc finger transcription factor ZIC3	—
							
Type I (+) SUMOAMVRs							
CASP8AP2 (Q9UKL3)	Gln1792Lys	K1792	ANRPLKCIVE**E**T YIDLTTESP	Experimental verification	—	Regulates proteasome-dependent degradation of FLASH/Casp8AP2	—
BHLHE41 (Q9C0J8)	Leu240 Lys	K240	DFLRCHEERI**L**R GHGADVKCV	Experimental verification	—	—	—
BHLHE42 (Q9C0J8)	Thr255Lys	K255	ADVKCVDWHP**T**K GLVVSGSKD	Experimental verification	—	—	—
NPM1 (P06748)	Arg101Lys	K101	GFEITPPVVL**R**L KCGSGPVHI	Experimental verification	—	Involve in the survival of the parasite	—

^a^Location and amino acid changes of variations in the proteins.

^b^Peptide sequences with 21-mer amino acids. The amino acids in the eleventh position with bold style and underline are sumoylated residues.

^c^The sources of the sumoylation sites.

^d^The sources of the mutations.

^e^The effects of the variations.

^f^Mendelian Inheritance in Man of the related disease.

**Table 4:** Several examples of the type II SUMOAMVR which include the amino acid variation not located on sumoylation position but on the adjacent positions that create (Type II (+)) or remove (Type II (−)) the sumoylation site.

Gene name (UniProtKB ID)	Variation site	Sumo- ylation site	Local peptide sequence^a^	Modify Source	Variation Source	Status	MIM
Type II (−) SUMOAMVRs							
ATXN1 (P54253)	Ser776Ala	K772	IEPSKPAATR**K**R RWSAPESRK	UniProtKB	dbSNP	DOID:1441- Spinocerebellar ataxia 1	164400
HSF1 (Q00613)	Ser303Ala	K298	PLSSSPLVRV**K**E EPPSPPQSP	UniProtKB	dbSNP	–	–
LMNA (P02545)	Gln203Gly	K201	VDAENRLQTM**K**E **E**LDFQKNIY	UniProtKB	dbSNP	DOID:11726- Emery-Dreifuss muscular dystrophy 3, autosomal recessive	181350 300696 310300 612998 612999 614302
USP25 (Q9UHP3)	Ile92Ala	K99	TNVIDLTGDD**K**D DLQRAIALS	UniProtKB	dbSNP	–	–
USP39 (Q53GS9)	Lys6Arg	K16	**K**RESRGSTRG**K**R ESESRGSSG	Experimental verification	–	DOID:10283- prostate cancer	176807 300147 300704 601518 602759

^a^Peptide sequences with 21-mer amino acids. The amino acids marked only with the bold style are variation sites, and those with bold style and underline are sumoylated residues.

**Table 5:** Several examples of the type III SUMOAMVR, caused by change in the types of E3 ligase involved, rather than in the sumoylation site itself, regardless of the positions of the variation.

Gene name (UniProtKB ID)	Variation site	Sumo- ylation site	Local peptide sequence	Modify Source	Variation Source	Status	MIM
Type III SUMOAMVRs							
CBX4 (O00257)	Thr497Ala	K494	AGEPPSSLQV**K**P E**T**PASAAVA	UniProtKB	dbSNP	Small decrease in ZNF131 sumoylation	–
TP53 (P04637)	Phe385Ala	K386	STSRH KKLM**FK**T EGPDSD	UniProtKB	dbSNP	Reduced SUMO1 conjugation DOID:1612-breast cancer	114480

**Table 6:** Statistical analysis of different types of SUMOAMVR based on the condition of disease-associated and polymorphic mutations affecting lysine sumoylation with 70% specificity option.

type	disease		polymorphism		unknown disease		p-value^c^
	num^a^	per.(%)^b^	num	per.(%)	num	per.(%)	
Type1(+)	69	0.28	138	0.36	28	0.43	0.09
Type1(−)	38	0.16	119	0.31	19	0.29	1.06E-4
Type11(+)	157	0.64	419	1.11	47	0.72	2.09E-9
Type11(−)	162	0.66	452	1.19	68	1.05	2.35E-9
Type111	752	3.09	2501	6.61	293	4.51	0.00
All	1178	4.83	3629	9.58	455	7.00	0.00

^a^The number of different types of SUMOAMVR.

^b^The proportion of different types of SUMOAMVR.

^c^The *P*-value of Pearson’s Chi-square test.

**Table 7:** Statistical comparison of the GO terms of the disease-related and normal sumoylation substrates.

Description of GO term	Diseased sumoylation		Normal sumoylation		P-value^c^	Over/Under^d^
	Num^a^	Per.(%)^b^	Num	Per.(%)		
**The most different biologic processes**						
Regulation of transcription from RNA polymeraseII promoter (GO:0006357)	49	7.62	89	26.89	2.51E-15	Over
Positive regulation of transcription (GO:0045941)	47	7.31	83	25.08	7.45E-14	Over
Positive regulation of gene expression (GO:0010628)	50	7.78	85	25.68	1.68E-13	Over
Negative regulation of nucleobase, nucleoside, and nucleic acid metabolic process (GO:0045934)	34	5.29	69	20.85	5.41E-13	Over
Positive regulation of nucleobase, nucleoside, and nucleic acid metabolic process (GO:0045935)	52	8.09	85	25.68	5.58E-13	Over
**The most different cellular compoents**						
Intracellular organelle lumen (GO:0070013)	105	16.33	135	40.79	2.18E-16	Over
Organelle lumen (GO:0043233)	110	17.11	136	41.09	1.44E-15	Over
Membrane-enclosedlumen (GO:0031974)	113	17.57	136	41.09	6.76E-15	Over
Cell projection (GO:0042995)	64	9.95	19	5.74	2.88E-02	Over
Axon (GO:0030424)	21	3.27	7	2.11	0.418431	Under
**The most different molecular functions**						
Sequence-specific DNA Binding (GO:0043565)	44	6.84	79	23.87	2.39E-05	Over
Transcription activator Activity (GO:0016563)	28	4.35	63	19.03	2.39E-05	Over
RNA polymerase II transcription factor activity (GO:0003702)	17	2.64	29	8.76	4.28E-04	Over
Structure-specific DNA binding (GO:0043566)	17	2.64	25	7.55	6.04E-04	Over
Double-stranded DNA binding (GO:0003690)	12	1.87	20	6.04	9.98E-04	Over

^a^The number of diseased sumoylation substrate in different GO terms.

^b^The proportion of diseased sumoylation substrate in different GO terms.

^c^The *P*-value of Fisher exact test (Two-sided category).

^d^Over - or under-representation of diseased sumoylation compared with normal sumoylation in different GO terms.
