# Supplementary material for: Systematic Analysis of the Genetic Variability That Impacts SUMO Conjugation and Their Involvement in Human Diseases
Source: Sci Rep. 2015 Jul 8;5:10900. doi: 10.1038/srep10900 (PMC4495600; doi:10.1038/srep10900)
Supplement: Supplementary Information [file srep10900-s1.doc]

**Supporting Information**

Systematic Analysis of the Genetic Variability That Impacts SUMO Conjugation and Their Involvement in Human Diseases

Hao-Dong Xu^1^, Shao-Ping Shi ^2^, Xiang Chen^1^, Jian-Ding Qiu^1, 3^*

^1^ Department of Chemistry, Nanchang University, Nanchang 330031, P.R.China

^2^ Department of Mathematics, Nanchang University, Nanchang 330031, P.R.China

^3^ Department of Materials and Chemical Engineering, Pingxiang College, Pingxiang 337055, P.R.China

^*^ To whom correspondence should be addressed. Tel: + 86 791 83969518; Email: [jdqiu@ncu.edu.cn](mailto:jdqiu@ncu.edu.cn)

**Table of Contents**

1. **Supplementary Experimental Procedures**

Ep1. Feature Extraction

Ep2. Structural characteristics

Ep3. Sequence-derived characteristics

Ep4. Evolutionary information

1. **Supplementary Tables**

Table S1 A summary of sumoylation site prediction tools.

Table S2. The possible examples of type I SUMOAMVR, which include the change of an amino acid with lysine residue or vice versa to create a potential new (Type I (+)) or remove an original lysine sumoylation site (Type I (-)).

Table S3. Several possible examples of the type II SUMOAMVR which include the amino acid variation not located on sumoylation position but on the adjacent positions that create (Type II (+)) or remove (Type II (-)) the sumoylation site.

Table S4. Several possible examples of the type III SUMOAMVR, caused by change in the types of E3 ligase Involved, rather than in the sumoylation site itself, regardless of the positions of the variation.

Table S5. Functional pathway analysis of disease-related sumoylation substrates.

Table S6. Several experimental examples of the SUMOAMVRs through our system.

Table S7. A summary of three type, annotation and dimensionality. Features can be classified into three major categories: sequence-derived features, structural characteristics, evolutionary information.

Table S8. The diagrammatic sumoylation motifs and their corresponding specific parameters of these motifs extracted from our dataset (background, all non-sumoylation sequence fragments and purple motifs are more representative of all seven motif).

Table SS1. The best 5 physicochemical properties used to shape SumoPred.

1. **Supplementary Figures**

Figure S1. ROC curves of different encoding after optimization based on the measurement of F-score using a 10-fold cross-validation strategy.（The curve of the red and blue represent features before and after optimization respectively）

Figure S2. ROC curves of the combination of multi-features after optimization based on the measurement of F-score using a 10-fold cross-validation strategy.（The curve of the red and blue represent features before and after optimization respectively）

Figure S3. sumoylation motifs and their corresponding sequence logos extracted from our dataset (background, all non-sumoylation sequence fragments).

Figure SS1. The average AASA value of residues around sumoylation sites and non-sumoylation sites

Figure SS2. The distribution of different secondary structure probability value of residues around sumoylation sites and non-sumoylation sites (photo on the left is the sumoylation sites distribution and right is non-sumoylation sites distribution)

Figure SS3. The average PSSM scores of residues around sumoylation sites and non-sumoylation sites

1. **Supplementary References**

**Supplemental experimental procedures**

**Feature Extraction**

**Structural** **characteristics**

*Average Accessible Surface Area (**AASA) features*

Pang et al.[^1^](#_ENREF_1) have investigated the structural environment of 8378 incidences of 44 types of PTMs and drew a conclusion that a side-chain of amino acid that undergoes posttranslational modification (PTM) prefers to be accessible on the surface of a protein. Therefore, the solvent accessibility of amino acid residues surrounding the sumoylation sites might be utilized when we distinguish the sumoylation site and non-sumoylation sites. In this part, we will demonstrate the effectiveness of AASA as features for sumoylation site prediction.

Figure SS1 plots the AASA formed from the 21-mer sumoylation sites in the constructed data set. On the basis of previous reports that “ΨKxE” might be the consensus motif that E3 ligase could recognize[^2^](#_ENREF_2), there is a considerable amount of large aliphatic branched hydrophobic amino acid, such as Leucine (L) and Valine(V), clustering at -1 position and Glutamic acid (E) at +2 position is the dominant residue, which resulting in AASA curve value in these positions showing relatively large differences between sumoylation sequences and non-sumoylation sequences and this trend is consistent with those data reported in the literature[^1^](#_ENREF_1). The fluctuant range of AASA of residues surrounding sumoylation sites is bigger than that of non-sumoylation sites. This indicates that the sumoylation processing might have occurred where the structural surroundings are relatively large variation range.

*Secondary Structure (SS)*

In addition explored the preference of the solvent accessible surface area that surrounds SUMO conjugation sites in protein tertiary structures, we also considered the preference of the secondary structure around the sumoylation sites. Since most of the experimentally verified sumoylated proteins do not have corresponding protein secondary structures in Protein Data Bank (PDB)[^3^](#_ENREF_3). Then the NetSurfP (Version 1.1) [^4^](#_ENREF_4) was utilized to compute the secondary structure that surrounds the sumoylation sites from the protein sequence. The distribution of different secondary structure probability value of residues around sumoylation sites and non-sumoylation sites was shown in the Figure SS2. Different colors of cylinder represent the incidence of helix, sheet and coil in different position of the fragment respectively. We found that it performed relatively large differences in the center site domains and especially at position -4 - +3, The probability value of sheet of sumoylated sequences are higher than non-sumoylation sequences while the probability value of helix exhibit opposite tendency. Due to differences in the performance of the underlying secondary structure, we then transform the three terms that specify the probability of secondary structure into numeric vectors to develop our model.

**Sequence-derived characteristics**

*The Composition of K-spaced Amino Acid Pairs (CKSAAP) Encoding*

In this paper, a sumoylation site is presented by a fragment of 21 residues with the residue lysine (K) in the central position. And CKSAAP encoding[^5^](#_ENREF_5) reflects the composition of k-spaced amino acid pairs (i.e., pairs that are separated by k other amino acids) within this sequence fragment. This feature vector is then adopted to represent the composition of these pairs and further to build our model, which can be described as follows:

（CAA CAC AAD …… COO )_441_

The value of each feature of CKSAAP encoding denotes the composition of the corresponding amino acid pair in the fragment. For example, if an AA pair appears m times in a sumoylation fragment, the composition of the AA pair in the vector (i.e., C_AA_) is equal to m. The amino acid pairs for k=0, 1… k_max_ are jointly considered in this study. Therefore, the total dimension of the proposed feature vector is 441×(k_max_+1). In our work, we define the k_max_ as equal to 4 when considering the dimension and overall performance

*AAindex*

Amino Acid index database (AAindex)[^6^](#_ENREF_6) contains a total of 544 amino acid indices, which includes a number of published indices that specify the physicochemical properties of amino acids. Therefore, according to the values associated with each physicochemical property, the amino acids adjacent to the sumoylated sites can be encoded as an input vector to develop our prediction model. And the amino acid indices with the value “NA” were replaced by 0 in this feature. We have examined all of the 544 physicochemical properties with the default parameters of SVM to investigate the contribution of each physicochemical property to build the model. Finally, 5 physicochemical properties were chosen from AAindex, as shown in TableSS1.

**Evolutionary information**

*Position Specific Scoring Matrix Profiles (PSSM)*

In biological analysis, one of the most important aspects of concern is the evolutionary conservation. The PSSM-encoding method has consistently been applied to predict biological problems because of its ability to reflect the evolutionary information of a sequence fragment, such as the subcellular localizations of Gram-negative bacterial proteins and prediction of RNA-binding sites and. Here, we used Position Specific Iterative BLAST (PSI-BLAST)[^9^](#_ENREF_9) to measure the conservation status for a specific residue. The PSSM conservation score were obtained by PSI-BLAST against whole Swiss-Prot protein database.

Figure SS3 summarizes the average PSSM scores formed from the 21-mer sumoylation sites and the 21-mer non-sumoylation sites in the constructed data set. We could found there are relatively large differences with respect to the values in different positions of sites between sumoylation sequences and non-sumoylation sequences and that the curve value of the sumoylated sequences at −1 position and +2 was extremely differential compare to that of the non-sumoylated sequences, which is conforming to previous reports that “ΨKxE” might be the consensus motif that E3 ligase could recognize[^2^](#_ENREF_2). This implied that the difference of evolutionary conservation between sumoylation and non-sumoylation sequences have a significant impact on prediction of sumoylation sites. In this regard, the prediction model was constructed with PSSM features.

**Supplementary Tables**

Table S1 A summary of sumoylation site prediction tools.

| Tool name | Web server;  Software Availability | Protein/ substrates | Training features | feature optimization | Web site |
| --- | --- | --- | --- | --- | --- |
| SUMOplot | Web server | -- | Only sequence | -- | http://www.abgent.com/tools/ |
| SUMOsp | Web server; | 144/239 | Only sequence | -- | http://bioinformatics.  lcd-ustc.org/sumosp/ |
| SUMOpre | No server | 268 | Only sequence | -- | -- |
| SSPFS | No server | 227 | Sequence + physicochemical  properties | -- | -- |
| SUMOsp 2.0 | Web server | 144/239 | Only sequence | -- | http://sumosp.biocuckoo.org/ |
| SeeSUMO | Web server | 263/457 | Only sequence | -- | http://bioinfo.ncu.edu.cn/ubiprober.aspx |
| SUMOhunt | No server | 452 | Sequence + physicochemical properties | -- | -- |
| SUMOhydro | Web server | 221/370 | Only sequence | -- | http://protein.cau.edu.cn/others/SUMOhydro/ |
| GPS-SUMO | Web server | 545/983 | Only sequence | Particle Swarm | http://sumosp.biocuckoo.org/ |
| SumoPred | Web server | 498/752 | Structure characteristics+  sequence-derived characteristics + evolutionary information | F-score | http://bioinfo.ncu.edu.cn/SUMOAMVR_Home.aspx |

Table S2. The possible examples of type I SUMOAMVR, which include the change of an amino acid with lysine residue or vice versa to create a potential new (Type I (+)) or remove an original lysine sumoylation site (Type I (-)). ^a^Location and amino acid changes of variations in the proteins. ^b^Peptide sequences with 21-mer amino acids. The amino acids in the eleventh position with bold style and underline are sumoylated residues. ^c^The effects of the variations. We referred to the feature tables of Swiss-Prot for these effects. ^d^Mendelian Inheritance in Man of the related disease.

| gene name | variation site^a^ | Sumo-  ylation  site | local peptide sequence^b^ | status^c^ | MIM^d^ | |
| --- | --- | --- | --- | --- | --- | --- |
| **Possible Type I (+) SUMOAMVR** | | | | | | |
| CAV3  (P56539) | VAR_021016  Asn33Lys | K33 | DLVNRDPKNI**N**E  DIVKVDFED | Disease:  Myopathy, distal, Tateyama type (MPDT)  ( rs1008642) | | 614321 |
| CBS  (P35520) | VAR_008076  Glu302Lys | K302 | ELNQTEQTTY**E**V  EGIGYDFIP | Disease:  Cystathionine beta-synthase deficiency (CBSD) | | 236200 |
| LMNA  (P02545) | VAR_009977  Asn195Lys | K195 | DEMLRRVDAE**N**R  LQTMKEELD | Disease:  Cardiomyopathy, dilated 1A (CMD1A)  （rs28933091） | | 115200 |
| ACTG1  (P63261) | VAR_067817  Thr203Lys | K203 | LTERGYSFTT**T**A  EREIVRDIK | Disease：  Baraitser-Winter syndrome 2 (BRWS2)  （rs281875327） | | 614583 |
| DYNC1H1  (Q14204) | VAR_067823  Glu1518Lys | K1518 | KLSPYYKVFE**E**D  ALSWEDKLN | Disease：  Mental retardation, autosomal dominant 13 (MRD13) | | 614563 |
| FLNA  (P21333) | VAR_015719  Thr555Lys | K555 | MVPGTYIVTI**T**W  GGQNIGRSP | Disease：  Otopalatodigital syndrome 2 (OPD2) | | 304120 |
| GCK  (P35557) | VAR_012351  Met210Lys | K210 | VAMVNDTVAT**M**I  SCYYEDHQC | Disease：  Maturity-onset diabetes of the young 2 (MODY2) | | 125851 |
| KRT75  (O95678) | VAR_038104  Glu337Lys | K337 | YEDIANRSRA**E**A  ESWYQTKYE | Disease：  Loose anagen hair syndrome (LAHS)  （rs2232398） | | 600628 |
| MAPT  (P10636) | VAR_010346  Asn596Lys | K596 | QPGGGKVQII**N**K  KLDLSNVQS | Disease：  Frontotemporal dementia (FTD) | | 600274 |

| **Possible Type I (-) SUMOAMVR** | | | | | |
| --- | --- | --- | --- | --- | --- |
| CBS  (P35520) | VAR_00802   | Lys102Gln | | --- | | K102 | INKIGKKFGL**K**C  ELLAKCEFF | Disease:  Cystathionine beta-synthase deficiency (CBSD)  （rs34040148） | 236200 |
| GJA1  (P17302) | VAR_015758  Lys102Asn | K102 | YLAHVFYVMR**K**EEKLNKKEEE | Disease:  Oculodentodigital dysplasia (ODDD) | 164200 |
| LMNA  (P02545) | VAR_039759  Lys97Glu | K97 | DARKTLDSVA**K**ERARLQLELS | Disease:  Cardiomyopathy, dilated 1A (CMD1A)  （rs59065411） | 115200 |
| STAT1  (P42224) | VAR_068713  Lys637Glu | K637 | DFHAVEPYTK**K**ELSAVTFPDI | Disease：  Mendelian susceptibility to mycobacterial disease (MSMD) | 209950 |
| ACADVL  (P49748) | VAR_000352  Lys382Gln | K382 | KIHNFGLIQE**K**LARMVMLQYV | Disease：  Acyl-CoA dehydrogenase very long-chain deficiency (ACADVLD)  （rs118204015） | 201475 |
| PTEN  (P60484) | VAR_008741  Lys289Glu | K289 | FIPGPEETSE**K**V  ENGSLCDQE | Disease：  Cowden syndrome 1 (CWS1) | 158350 |
| THRB  (P10828) | VAR_004651  Lys443Glu | K443 | ACHASRFLHM**K**VECPTELFPP | Disease：  Generalized thyroid hormone resistance (GTHR) | 188570 |
| MAPT  (P10636) | VAR_010344  Lys574Thr | K574 | PVPMPDLKNV**K**SKIGSTENLK | Disease：  Pick disease of the brain (PIDB) | 172700 |

Table S3. Several possible examples of the type II SUMOAMVR which include the amino acid variation not located on sumoylation position but on the adjacent positions that create (Type II (+)) or remove (Type II (-)) the sumoylation site. ^a^Peptide sequences with 21-mer amino acids. The amino acids marked only with the bold style are variation sites, and those with bold style and underline are sumoylated residues.

| gene name | variation site | Sumo-  ylation  site | local peptide sequence^a^ | status | MIM |
| --- | --- | --- | --- | --- | --- |
| **Possible Type II (+) SUMOAMVR** | | | | | |
| AR  (P10275) | VAR_00988  Leu812Phe | K808 | QITPQEFLCM**K**ALL**L**FSIIPV | Disease:  Androgen insensitivity syndrome (AIS) | 300068 |
| CAV3  (P56539) | VAR_043699  Ser141Arg | K149 | VC**S**SIKVVLR**K**EV | Disease:  Long QT syndrome 9 (LQT9) | 611818 |
| LMNA  (P02545) | VAR_070175  Glu138Lys | K144 | LKDLEALLNS**K**EALS**T**ALSE | Disease:  Hutchinson-Gilford progeria syndrome (HGPS) | 176670 |
| STAT1  （P42224） | VAR_065937  Cys174Arg | K173 | LEDLQDEYDF**KC**KTLQNREHE | Disease:  Candidiasis, familial, 7 (CANDF7) | 614162 |
| WT1  （P19544） | VAR_043811  His401Tyr | K391 | KPFQCKTCQR**K**FSRSDHLKT**H** | Disease:  Denys-Drash syndrome (DDS) | 194080 |
| BUB1B  （O60566） | VAR_028924  Arg814His | K812 | PWDFYINLKL**K**E**R**LNEDFDHF | Disease:  Mosaic variegated aneuploidy syndrome 1 (MVA1)  （rs28989182） | 257300 |
| CHD7  （Q9P2D1） | VAR_033247  Leu1294Pro | K1303 | V**L**IDKLLPKL**K**AGGHRVLIFS | Disease:  CHARGE syndrome (CHARGES) | 214800 |
| ESR1  （P033720 | VAR_004672  Val364Glu | K362 | RELVHMINWA**K**R**V**PGFVDLTL | Disease:  Estrogen resistance (ESTRR) | 615363 |
| GCK  (P35557) | VAR_012350  Leu164Pro | K172 | GI**L**LNWTKGF**K**ASGAEGNNVV | Dise Maturity-onset diabetes of the young 2 (MODY2)ase: | 125851 |
| NIN  (Q8N4C6) | VAR_069084  Asn1709Ser | K1714 | SVLSY**N**EKLL**K**EKEALSEELN | Disease:  Seckel syndrome 7 (SCKL7) | 614851 |
| PTEN  (P60484) | VAR_032634  His93Arg | K102 | D**H**NPPQLELI**K**PFCEDLDQWL | Disease:  Macrocephaly/autism syndrome (MCEPHAS) | 605309 |
| RB1  (P06400) | VAR_011581  Lys616Glu | K614 | DMYLSPVRSP**K**K**K**GSTTRVNS | Disease:  Childhood cancer retinoblastoma (RB) | 180200 |
| ATP2A2  (P16615) | VAR_008609  Asn39Thr | K33 | GLSLEQVKKL**K**ERWGS**N**ELPA | Disease:  Darier disease (DD) | 124200 |
| SOD1  (P00441) | VAR_013525  Phe46Cys | K37 | NGPVKVWGSI**K**GLTEGLHG**F**H | Disease:  Amyotrophic lateral sclerosis 1 (ALS1) | 105400 |
| **Possible Type II (-) SUMOAMVR** | | | | | |
| AR  (P10275) | VAR_013480  Asp879Tyr | K883 | LHQFTF**D**LLI**K**S  HMVSVDFPE | Disease:  Androgen insensitivity syndrome (AIS) | 300068 |
| CBS  (P35520) | VAR_008049  Pro49Leu | K39 | EKGSPEDKEA**K**EPLWIRPDA**P** | Disease:  Cystathionine beta-synthase deficiency (CBSD) | 236200 |
| GJA1  (P17302) | VAR_059003  Glu110Asp | K108 | YVMRKEEKLN**K**K**E**EELKVAQT | Disease:  Oculodentodigital dysplasia (ODDD) | 164200 |
| FOXL2  (P17302) | VAR_021196  Ser58Leu | K48 | GGGGGGTAPE**K**PDPAQKPPY**S** | Disease:  Blepharophimosis, ptosis, and epicanthus inversus syndrome (BPES) | 110100 |
| LMNA  (P02545) | VAR_070177  Ile210Ser | K201 | VDAENRLQTM**K**EELDFQKN**I**Y | Disease:  Cardiomyopathy, dilated 1A (CMD1A) | 115200 |
| IKBKG  (Q9Y6K9) | VAR_031960  Arg319Gln | K309 | PVLKAQADIY**K**ADFQAERQA**R** | Disease:  X-linked familial atypical micobacteriosis 1 (AMCBX1) | 300636 |
| PARK7  (Q99497) | VAR_020493  Glu64Asp | K62 | ICPDASLEDA**K**K**E**GPYDVVVL | Disease:  Parkinson disease 7 (PARK7) | 606324 |
| PDE4D  (Q08499) | VAR_069453  Glu590Ala | K586 | SKHMNLLADL**K**TMV**E**TKKVTS | Disease:  Acrodysostosis 2, with or without hormone resistance (ACRDYS2) | 614613 |
| NR5A1  (Q13285) | VAR_063257  Gly91Ser | K100 | GGRNKFGPMY**K**RDRALKQQ**K**K | Disease:  46,XY sex reversal 3 (SRXY3) | 612965 |
| ACTB  (P60709) | VAR_067810  Asn12Asp | K18 | LVVD**N**GSGMC**K**AGFAGDDAPR | Disease:  Baraitser-Winter syndrome 1 (BRWS1)  (rs281875331) | 243310 |

Table S4. Several possible examples of the type III SUMOAMVR, caused by change in the types of E3 ligase Involved, rather than in the sumoylation site itself, regardless of the positions of the variation.

| gene name | variation site | Sumo-  ylation  site | local peptide sequence | status | MIM |
| --- | --- | --- | --- | --- | --- |
| **Possible Type III SUMOAMVR** | | | | | |
| THRB  P10828 | VAR_059041  Ala268Gly | K263 | APIVNAPEGG**K**V  DLE**A**FSHFT | Disease:  Generalized thyroid hormone resistance (GTHR) | 188570 |
| VHL  P40337 | VAR_034998  Leu163Pro | K171 | RC**L**QVVRSLV**K**P  ENYRRLDIV | Disease:  Renal cell carcinoma (RCC)  (rs28940297) | 144700 |
| WRN  Q14191 | VAR_026589  Lys135Glu | K134 | LKMLLENKAV**KK**AGVGIEGDQ | Disease:  Werner syndrome (WRN) | 277700 |
| APP  P05067 | VAR_044424  Asp678Asn | K670 | NIKTEEISEV**K**M  DAEFRH**D**SG | Disease:  Alzheimer disease 1 (AD1) | 104300 |
| MEN1  O00255 | VAR_005425  Pro12Leu | K4 | MGLKAAQ**K**TLF**P**LR | Disease:  Familial multiple endocrine neoplasia type I (MEN1) | 131100 |
| PRDM16  Q9HAZ2 | VAR_070212  Glu271Lys | K269 | ALYEGLAEEL**K**P  **E**GLGGGSGQ | Disease:  Cardiomyopathy, dilated 1LL (CMD1LL) | 615373 |
| MAPT  P10636 | VAR_010346  Asn596Lys | K598 | GGGKVQII**N**K**K**L  DLSNVQSKC | Disease:  Frontotemporal dementia (FTD) | 600274 |

Table S5. Functional pathway analysis of disease-related sumoylation substrates.

| Term | Count | per.(%) | P-Value | Genes | FDR |
| --- | --- | --- | --- | --- | --- |
| hsa05200:Pathways in cancer | 45 | 7.00 | 3.61E-07 | 796931, 779920, 791692, 802006, 821298, 824925, 783175, 776434, 790888, 797411, 775941, 781513, 815476, 819591, 796239, 794116, 796732, 775183, 780063, 781622, 775481, 776910, 782170, 810969, 772977, 788864, 797705, 797532, 812548, 796320, 811245, 787522, 784752, 778156, 812931, 802140, 800291, 813763, 819422, 826200, 785016, 811347, 804645, 793566, 800275 | 0.00 |
| hsa05412:Arrhythmogenic right ventricular cardiomyopathy (ARVC) | 15 | 2.33 | 1.01E-06 | 787863, 819220, 795462, 823421, 783175, 784677, 783908, 806627, 798372, 777044, 795592, 810858, 782027, 811347, 821312 | 0.03 |
| hsa05215:Prostate cancer | 18 | 2.80 | 1.68E-06 | 779920, 791692, 821298, 776910, 772977, 781513, 815476, 788864, 797705, 797532, 794116, 796239, 811245, 812931, 802140, 796732, 813763, 785016 | 0.04 |
| hsa05212:Pancreatic cancer | 14 | 2.18 | 1.87E-06 | 791692, 821298, 824925, 810969, 781513, 788864, 797705, 797532, 796320, 811245, 812931, 796732, 813763, 775183 | 0.08 |
| hsa05220:Chronic myeloid leukemia | 17 | 2.64 | 6.41E-06 | 791692, 821298, 772977, 781513, 775941, 788864, 797705, 797532, 796320, 796239, 811245, 812931, 802140, 796732, 813763, 775183, 785016 | 0.10 |
| hsa05222:Small cell lung cancer | 15 | 2.33 | 2.19E-05 | 779920, 791692, 783175, 788864, 797705, 797532, 819591, 811245, 812931, 802140, 796732, 800291, 811347, 804645, 800275 | 0.10 |
| hsa05410:Hypertrophic cardiomyopathy (HCM) | 19 | 2.95 | 2.55E-05 | 795462, 783175, 784677, 783908, 782130, 790888, 809025, 790229, 809566, 817943, 806627, 807536, 808632, 795592, 777044, 810858, 782027, 811347, 821312 | 0.12 |
| hsa05414:Dilated cardiomyopathy | 19 | 2.95 | 2.86E-05 | 795462, 783175, 784677, 783908, 782130, 790888, 809025, 790229, 809566, 817943, 806627, 807536, 808632, 795592, 777044, 810858, 782027, 811347, 821312 | 0.12 |
| hsa04510:Focal adhesion | 32 | 4.98 | 3.74E-05 | 779920, 791692, 821298, 795569, 783175, 776434, 799424, 781513, 819591, 778146, 796732, 780010, 795462, 779750, 772977, 786593, 787944, 788864, 797705, 815273, 797532, 777044, 800291, 781103, 777404, 785016, 811347, 808600, 821312, 804645, 803177, 800275 | 0.15 |
| hsa04623:Cytosolic DNA-sensing pathway | 13 | 2.02 | 4.17E-05 | 819508, 811245, 802140, 776434, 781513, 775941, 788864, 797705, 797532, 796239, 796732, 813763, 785016 | 0.35 |
| hsa04142:Lysosome | 25 | 3.89 | 4.58E-05 | 791081, 794164, 784989, 793306, 785820, 777657, 818077, 805340, 786704, 808018, 794960, 776708, 790712, 779579, 823216, 807305, 809467, 794348, 813374, 809039, 799924, 784322, 818586, 804338, 826221 | 0.40 |
| hsa05213:Endometrial cancer | 15 | 2.33 | 6.05E-05 | 779920, 791692, 821298, 775481, 772977, 781513, 788864, 797705, 797532, 796239, 784752, 796732, 813763, 826200, 785016 | 0.41 |
| hsa04512:ECM-receptor interaction | 18 | 2.80 | 7.85E-05 | 780010, 795569, 795462, 783175, 779750, 799424, 805324, 787944, 819591, 820427, 800291, 781103, 777404, 811347, 808600, 804645, 800275, 803177 | 0.46 |
| hsa04610:Complement and coagulation cascades | 15 | 2.33 | 3.17E-04 | 809273, 788845, 822080, 820621, 801450, 799424, 811407, 789994, 813860, 794018, 803958, 806146, 773626, 815415, 796887 | 0.49 |
| hsa05223:Non-small cell lung cancer | 13 | 2.02 | 3.37E-04 | 791692, 821298, 772977, 776434, 781513, 788864, 797705, 797532, 796239, 812931, 796732, 813763, 785016 | 0.51 |
| hsa05214:Glioma | 14 | 2.18 | 4.29E-04 | 779920, 791692, 821298, 772977, 776434, 781513, 788864, 797705, 797532, 796239, 812931, 796732, 813763, 785016 | 0.53 |
| hsa05221:Acute myeloid leukemia | 13 | 2.02 | 5.77E-04 | 791692, 821298, 772977, 781513, 788864, 797705, 797532, 796239, 811245, 796732, 813763, 785016, 793566 | 0.71 |
| hsa05210:Colorectal cancer | 16 | 2.49 | 8.48E-04 | 791692, 821298, 775481, 772977, 781513, 788864, 797705, 797532, 812548, 796320, 778156, 784752, 796732, 813763, 826200, 775183 | 1.04 |
| hsa05211:Renal cell carcinoma | 14 | 2.18 | 1.25E-03 | 796931, 791692, 821298, 802006, 772977, 781513, 788864, 797705, 797532, 796239, 814267, 796732, 813763, 785016 | 1.53 |
| hsa00640:Propanoate metabolism | 9 | 1.40 | 1.68E-03 | 794504, 786562, 823298, 774315, 808472, 789097, 820238, 786092, 812798 | 2.05 |
| hsa00531:Glycosaminoglycan degradation | 7 | 1.09 | 3.12E-03 | 808018, 794348, 793306, 804338, 790712, 785820, 805340 | 3.77 |
| hsa00280:Valine, leucine and isoleucine degradation | 10 | 1.56 | 3.90E-03 | 825062, 794504, 786562, 823298, 817979, 774315, 808472, 819508, 811245, 802140 | 4.69 |
| hsa05216:Thyroid cancer | 8 | 1.24 | 4.05E-03 | 780063, 821298, 796239, 782170, 797411, 790888, 813763, 785016 | 4.87 |
| hsa00511:Other glycan degradation | 6 | 0.93 | 4.55E-03 | 807305, 818586, 784989, 793306, 790712, 805340 | 5.46 |
| hsa05218:Melanoma | 13 | 2.02 | 4.88E-03 | 779920, 791692, 821298, 781513, 815476, 788864, 797705, 797532, 796239, 812931, 796732, 813763, 785016 | 5.84 |
| hsa00071:Fatty acid metabolism | 9 | 1.40 | 6.28E-03 | 821107, 794504, 783247, 808472, 789097, 803859, 820238, 812798, 777513 | 7.46 |
| hsa04662:B cell receptor signaling pathway | 13 | 2.02 | 6.86E-03 | 791692, 824956, 772977, 781513, 788864, 797705, 797532, 796239, 811245, 802140, 796732, 813763, 785016 | 8.12 |
| hsa04260:Cardiac muscle contraction | 13 | 2.02 | 7.64E-03 | 784677, 782130, 790888, 790229, 809566, 806627, 817943, 807536, 808632, 772604, 816702, 810858, 782027 | 9.01 |
| hsa04910:Insulin signaling pathway | 19 | 2.95 | 7.68E-03 | 791692, 821298, 810832, 772977, 781513, 775941, 788864, 797705, 781273, 797532, 796239, 805402, 787161, 796732, 813763, 785016, 792841, 793730, 815222 | 9.05 |
| hsa04950:Maturity onset diabetes of the young | 7 | 1.09 | 7.92E-03 | 798450, 787456, 787161, 820215, 816853, 793730, 789275 | 9.33 |
| hsa04960:Aldosterone-regulated sodium reabsorption | 9 | 1.40 | 8.58E-03 | 797532, 776434, 816702, 772604, 776650, 813763, 797705, 815222, 801280 | 10.06 |
| hsa00860:Porphyrin and chlorophyll metabolism | 8 | 1.24 | 8.61E-03 | 797496, 818050, 811506, 818834, 801793, 796107, 780311, 779667 | 10.09 |
| hsa00650:Butanoate metabolism | 8 | 1.24 | 8.61E-03 | 773505, 818970, 799462, 794862, 808472, 789097, 820238, 812798 | 10.09 |
| hsa04370:VEGF signaling pathway | 12 | 1.87 | 1.61E-02 | 797532, 791692, 796239, 810824, 776434, 796732, 781513, 813763, 821973, 785016, 788864, 797705 | 18.14 |
| hsa04664:Fc epsilon RI signaling pathway | 12 | 1.87 | 1.95E-02 | 797532, 791692, 796239, 810824, 824956, 772977, 796732, 781513, 813763, 785016, 788864, 797705 | 21.51 |
| hsa00020:Citrate cycle (TCA cycle) | 7 | 1.09 | 1.95E-02 | 796931, 775108, 799462, 794862, 805596, 809354, 786092 | 21.52 |
| hsa00010:Glycolysis / Gluconeogenesis | 10 | 1.56 | 2.62E-02 | 787439, 815605, 799462, 794862, 787161, 813329, 792841, 793730, 808472, 820238 | 27.92 |
| hsa00970:Aminoacyl-tRNA biosynthesis | 8 | 1.24 | 2.74E-02 | 793517, 791126, 815325, 778056, 795407, 796107, 802774, 787847 | 28.97 |
| hsa00230:Purine metabolism | 19 | 2.95 | 2.94E-02 | 790167, 801948, 810430, 788123, 819852, 785205, 803232, 790179, 786637, 782508, 817849, 807165, 793807, 805413, 808179, 800322, 806759, 798967, 793730 | 30.74 |
| hsa05219:Bladder cancer | 8 | 1.24 | 3.09E-02 | 804719, 821298, 796239, 787522, 812931, 781513, 813763, 785016 | 32.05 |
| hsa00052:Galactose metabolism | 6 | 0.93 | 3.21E-02 | 787161, 776708, 809039, 792841, 776167, 805340 | 33.11 |
| hsa04722:Neurotrophin signaling pathway | 16 | 2.49 | 3.37E-02 | 791692, 821298, 782170, 772977, 781513, 788864, 797705, 816836, 797532, 796239, 813199, 802140, 796732, 813763, 801005, 785016 | 34.45 |
| hsa00520:Amino sugar and nucleotide sugar metabolism | 8 | 1.24 | 3.88E-02 | 815605, 826287, 793306, 787161, 790712, 809887, 776167, 823205 | 38.52 |
| hsa00290:Valine, leucine and isoleucine biosynthesis | 4 | 0.62 | 4.13E-02 | 793517, 799462, 794862, 787847 | 40.49 |
| hsa04010:MAPK signaling pathway | 28 | 4.35 | 4.51E-02 | 791692, 821298, 810824, 776434, 781513, 815476, 816836, 796239, 778146, 796732, 801005, 775183, 821973, 813774, 776910, 782170, 784677, 772977, 786593, 788864, 787522, 811245, 813763, 813479, 819422, 785016, 809288, 810858 | 43.29 |
| hsa04660:T cell receptor signaling pathway | 14 | 2.18 | 4.73E-02 | 791692, 772977, 781513, 775941, 788864, 797705, 797532, 810315, 796239, 811245, 802140, 796732, 813763, 785016 | 44.93 |
| hsa04730:Long-term depression | 10 | 1.56 | 5.37E-02 | 788685, 821298, 796239, 810824, 778449, 776434, 781513, 813763, 785016, 809288 | 49.27 |
| hsa00500:Starch and sucrose metabolism | 7 | 1.09 | 6.19E-02 | 815605, 801948, 805402, 787161, 776708, 792841, 780311 | 54.42 |
| hsa00620:Pyruvate metabolism | 7 | 1.09 | 6.85E-02 | 799462, 794862, 805596, 793730, 808472, 820238, 812798 | 58.24 |
| hsa00410:beta-Alanine metabolism | 5 | 0.78 | 7.24E-02 | 794504, 815908, 808472, 789097, 820238 | 60.34 |
| hsa04150:mTOR signaling pathway | 8 | 1.24 | 7.60E-02 | 797532, 791692, 821298, 810832, 796732, 801005, 788864, 797705 | 62.19 |
| hsa00603:Glycosphingolipid biosynthesis | 4 | 0.62 | 7.75E-02 | 793306, 794960, 809039, 790712 | 62.93 |
| hsa03430:Mismatch repair | 5 | 0.78 | 8.28E-02 | 812548, 793807, 822662, 826200, 778843 | 65.48 |

Table S6. Several experimental examples of the SUMOAMVRs through our system.


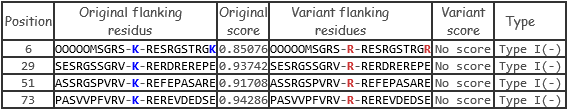


Table S7. A summary of three type, annotation and dimensionality. Features can be classified into three major categories: sequence-derived features, structural characteristics, evolutionary information.

| Feature type | Annotation | The total number of dimension before optimization | The total number of dimension after optimization |
| --- | --- | --- | --- |
| sequence-derived features | The Composition of K-spaced Amino Acid Pairs (CKSAAP) | 2205 | 255 |
|  | AAindex | 105 | 105 |
| structure features | Average Accessible Surface Area (AASA) | 21 | 2 |
|  | Secondary Structure (SS) | 63 | 26 |
| evolutionary information of amino acids | Position Specific Scoring Matrix Profiles (PSSM) | 441 | 13 |
| Total | 5 | 2835 | 401 |

Table S8. The diagrammatic sumoylation motifs and their corresponding specific parameters of these motifs extracted from our dataset (background, all non-sumoylation sequence fragments and purple motif is more representative of all seven motif).


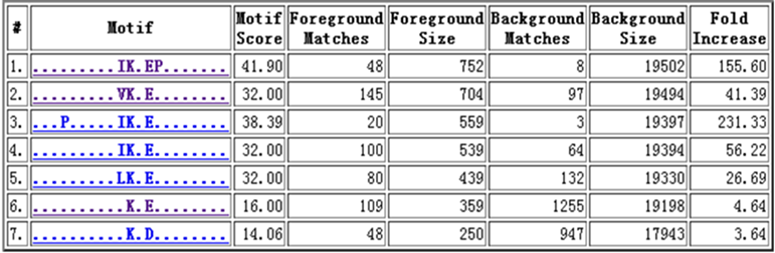


Table SS1. The best 5 physicochemical properties utilized to shape SumoPred.

| AAindex name | AAindex ID | References |
| --- | --- | --- |
| Polar requirement | WOEC730101 | Woese, 1973 |
| Normalized frequency of Bata-structure | NAGK730102 | Nagano, 1973 |
| Dependence of partition coefficient on ionic strength | ZASB820101 | Zaslavsky et al.,  1982 |
| Normalized frequency of beta-sheet from LG | PALJ810103 | Palau et al., 1981 |
| Surface composition of amino acids in intracellular proteins of mesophylls | FUKS010102 | Fukuchi-Nishikawa, 2001 |


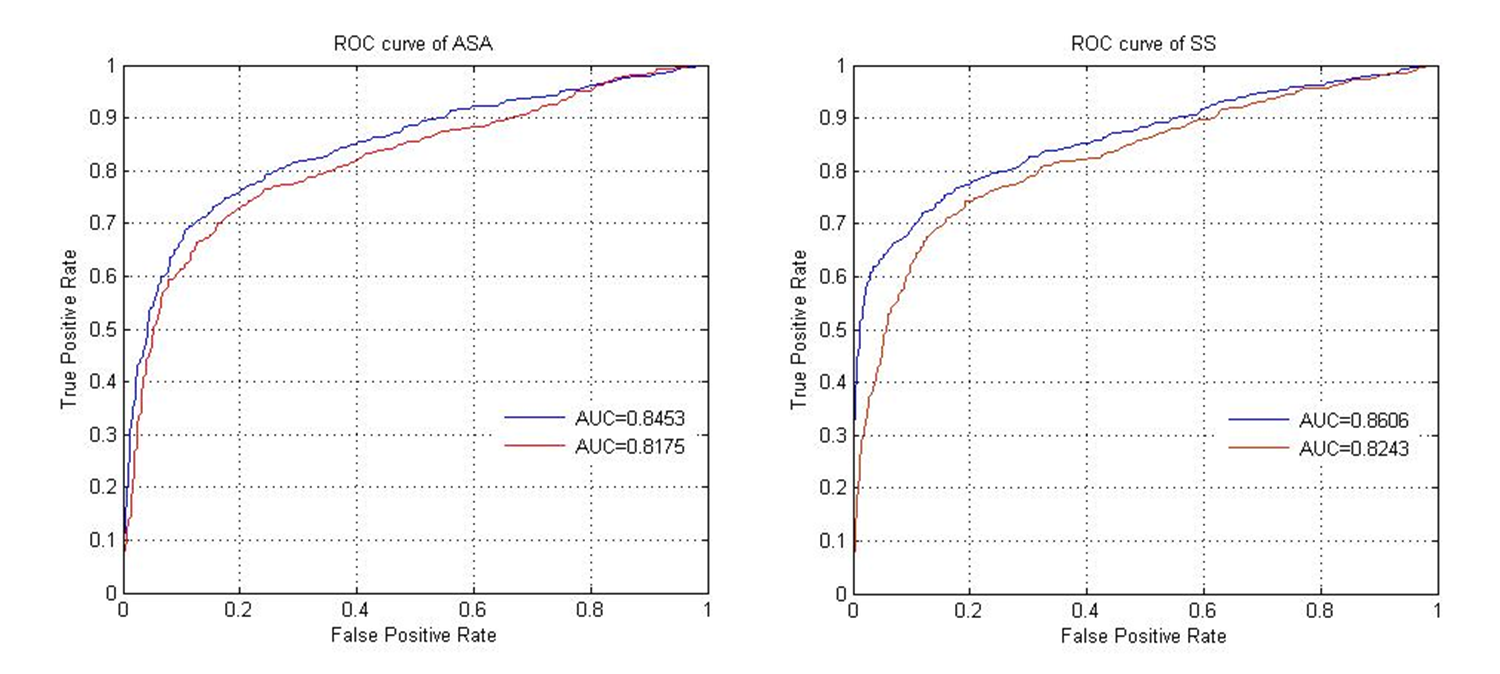

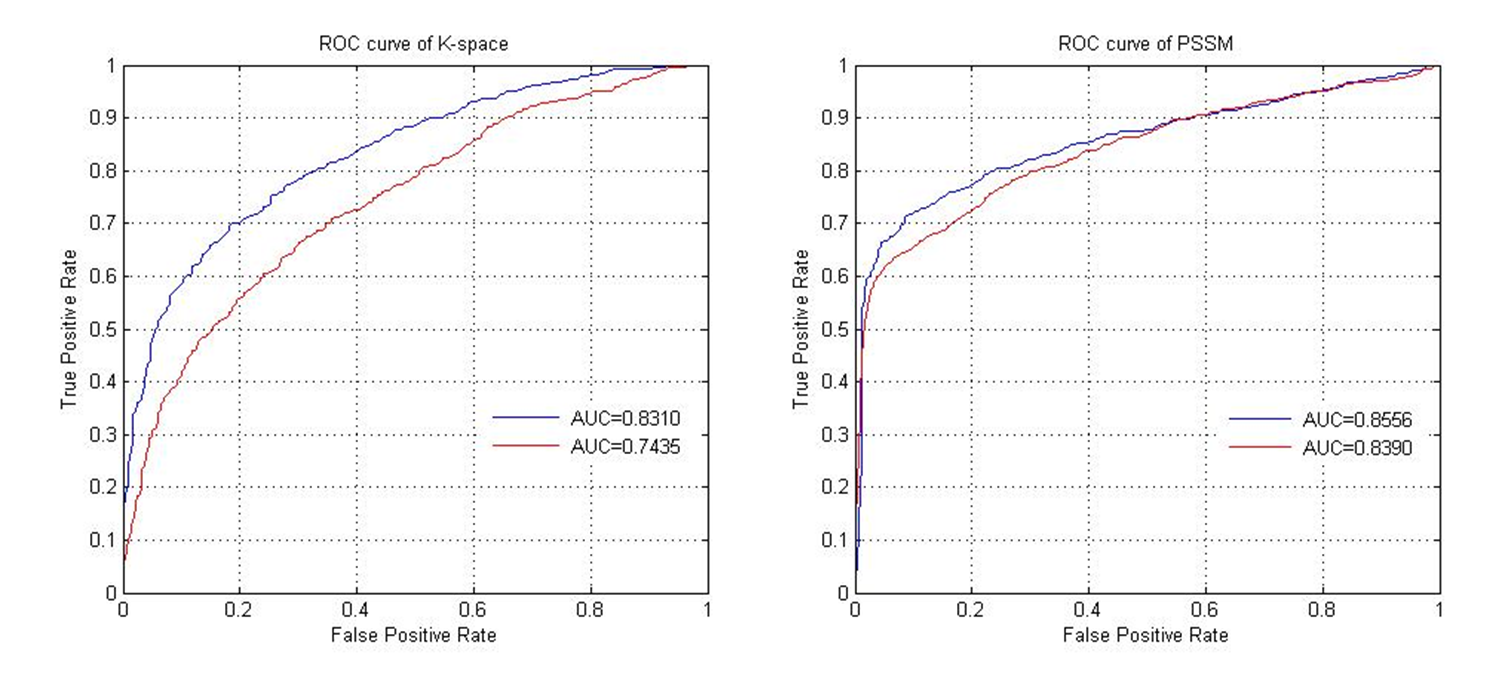
**Supplementary Figure**

Figure S1.ROC curves of different encoding after optimization based on the measurement of F-score using a 10-fold cross-validation strategy.（The curve of the red and blue represent features before and after optimization respectively）


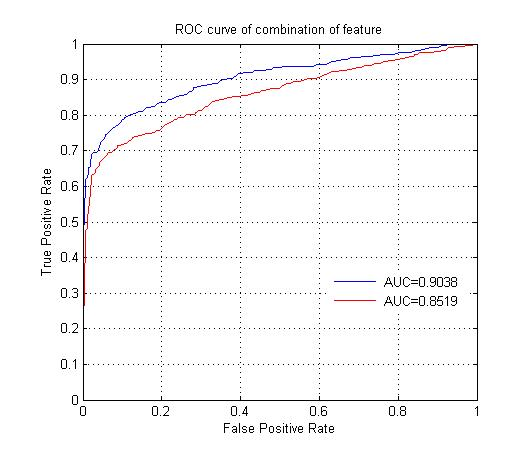


Figure S2.ROC curves of the combination of multi-features after optimization based on the measurement of F-score using a 10-fold cross-validation strategy.（The curve of the red and blue represent features before and after optimization respectively）


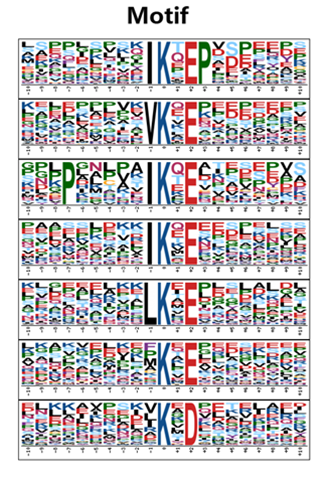


Figure S3. sumoylation motifs and their corresponding sequence logos extracted from our dataset (background, all non-sumoylation sequence fragments).


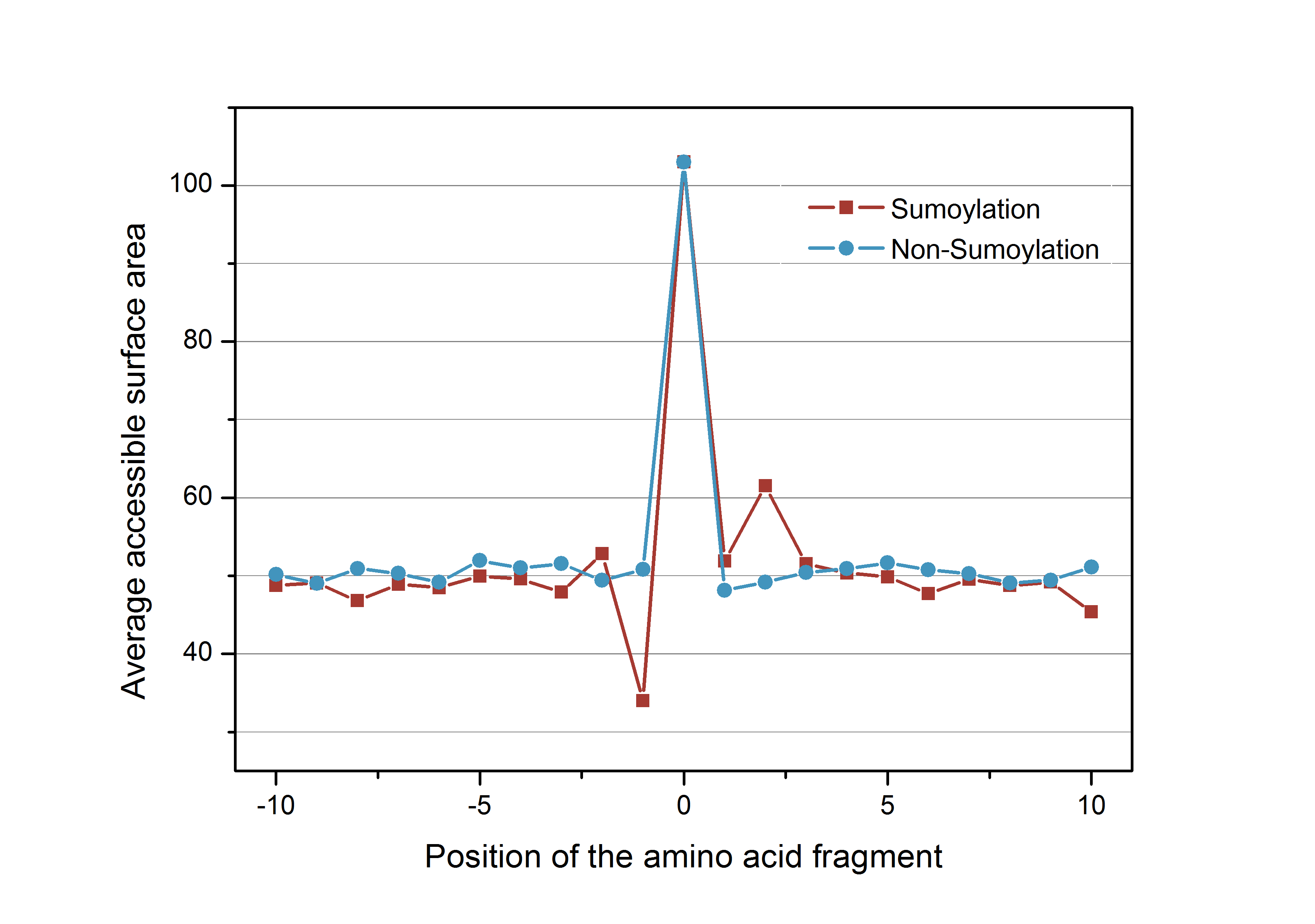


Figure SS1.The average AASA value of residues around sumoylation sites and non-sumoylation sites


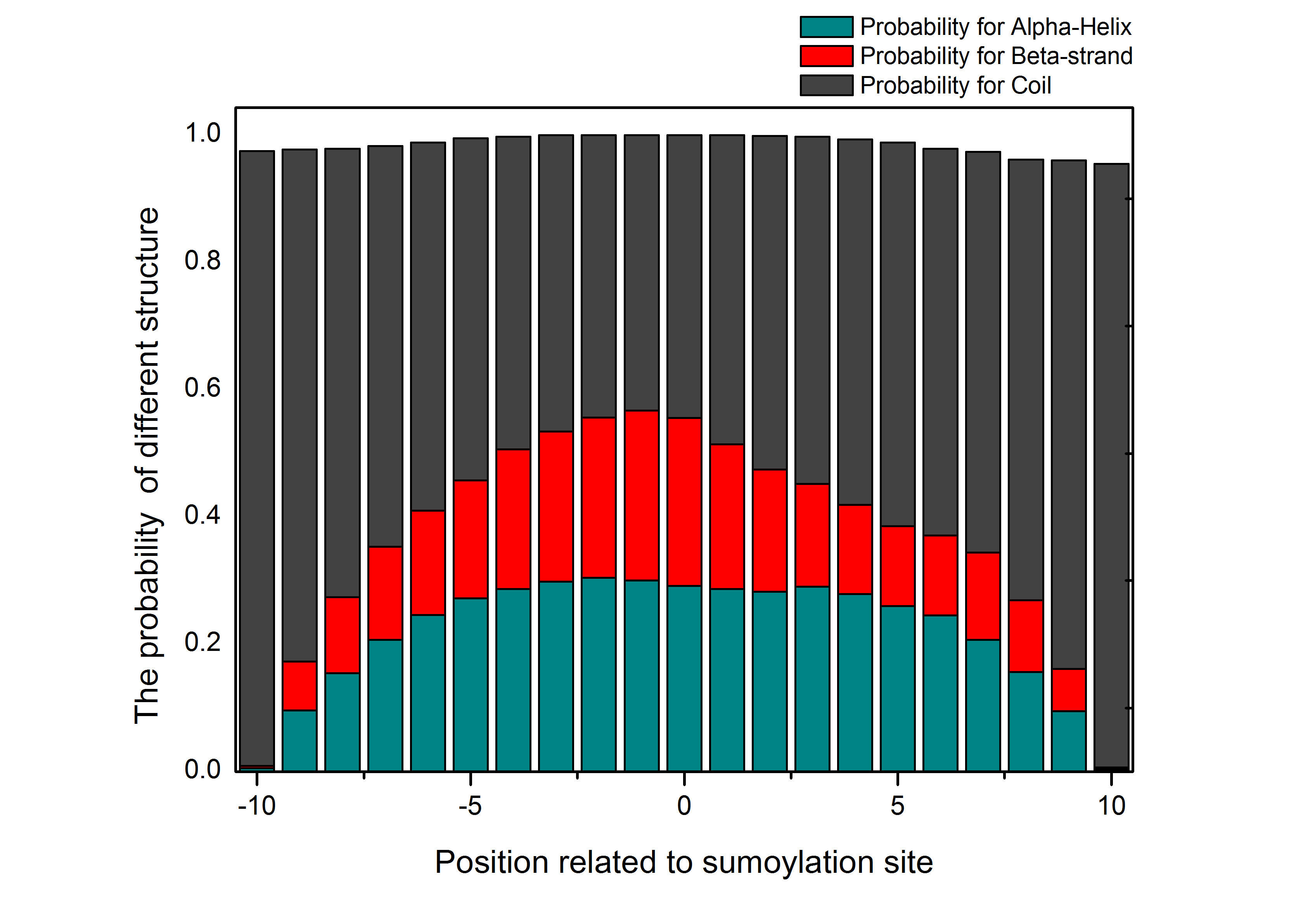

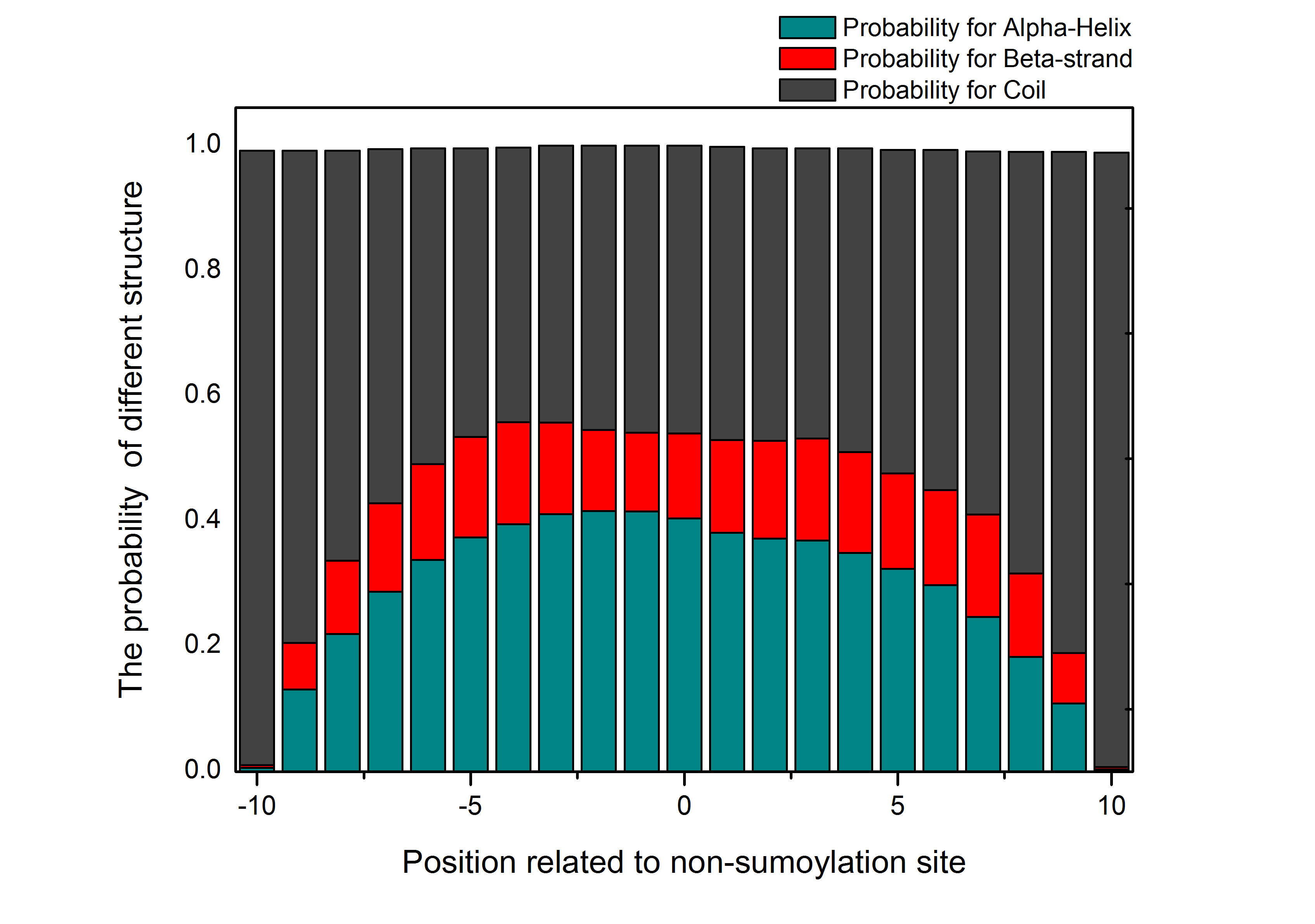


Figure SS2. The distribution of different secondary structure probability value of residues around sumoylation sites and non-sumoylation sites (photo on the left is the sumoylation sites distribution and right is non-sumoylation sites distribution)


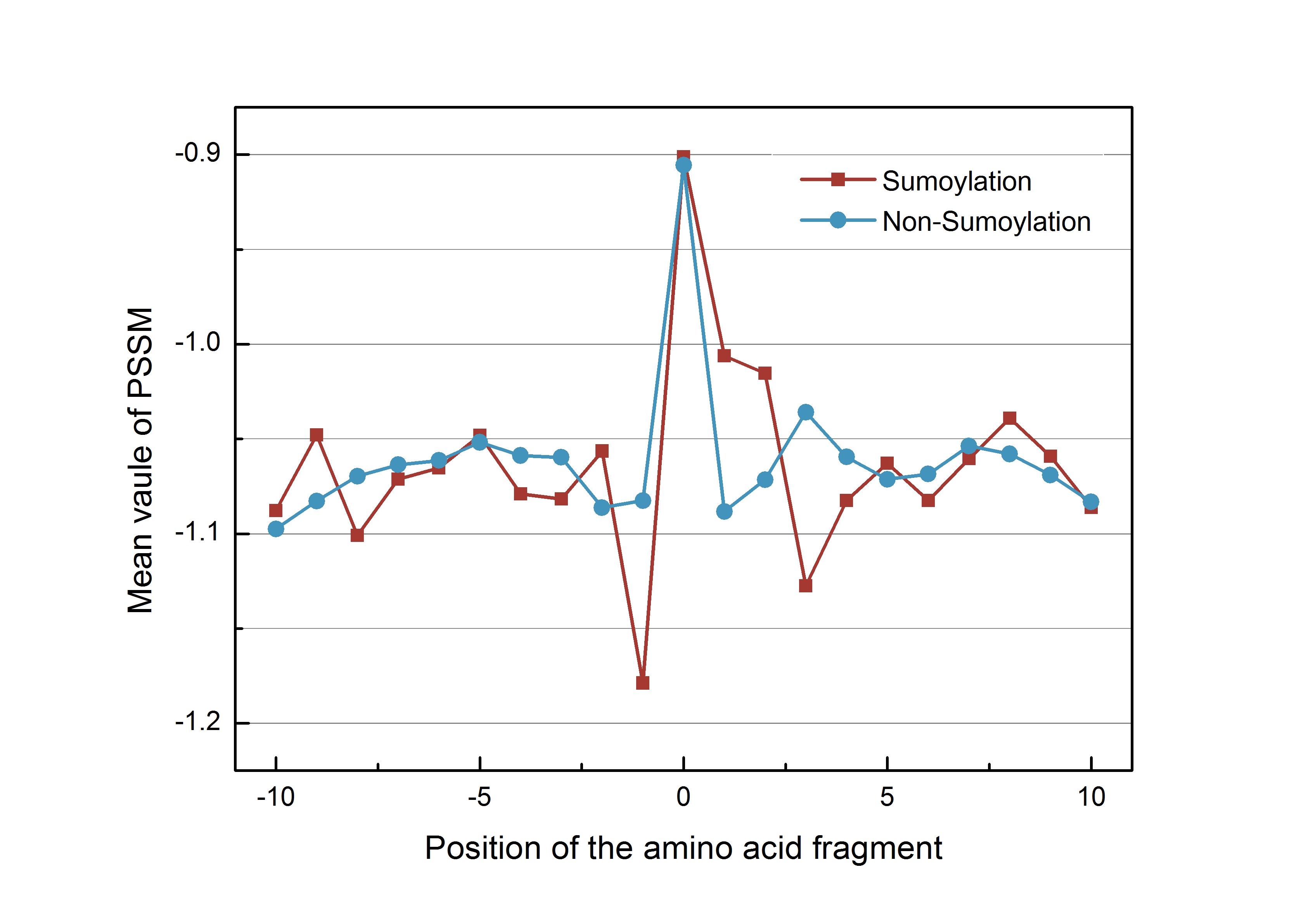


Figure SS3.The average PSSM scores of residues around sumoylation sites and non-sumoylation sites.

**Supplementary References**

1 Pang, C. N. I., Hayen, A. & Wilkins, M. R. Surface accessibility of protein post-translational modifications. *Journal of Proteome Research* **6**, 1833-1845, (2007).

2 Yang, S.-H., Galanis, A., Witty, J. & Sharrocks, A. D. An extended consensus motif enhances the specificity of substrate modification by SUMO. *Embo Journal* **25**, 5083-5093, (2006).

3 Berman, H. M. *et al.* The Protein Data Bank. *Nucleic Acids Research* **28**, 235-242, (2000).

4 Petersen, B., Petersen, T. N., Andersen, P., Nielsen, M. & Lundegaard, C. A generic method for assignment of reliability scores applied to solvent accessibility predictions. *Bmc Structural Biology* **9**, (2009).

5 Chen, Y.-Z., Chen, Z., Gong, Y.-A. & Ying, G. SUMOhydro: A Novel Method for the Prediction of Sumoylation Sites Based on Hydrophobic Properties. *Plos One* **7**, (2012).

6 Kawashima, S. *et al.* AAindex: amino acid index database, progress report 2008. *Nucleic Acids Research* **36**, D202-D205, (2008).

7 Cheng, C.-W., Su, E. C.-Y., Hwang, J.-K., Sung, T.-Y. & Hsu, W.-L. Predicting RNA-binding sites of proteins using support vector machines and evolutionary information. *Bmc Bioinformatics* **9**, (2008).

8 Kumar, M., Gromiha, A. M. & Raghava, G. P. S. Prediction of RNA binding sites in a protein using SVM and PSSM profile. *Proteins-Structure Function and Bioinformatics* **71**, 189-194, (2008).

9 Altschul, S. F. *et al.* Gapped BLAST and PSI-BLAST: a new generation of protein database search programs. *Nucleic Acids Research* **25**, 3389-3402, (1997).
